# Neoadjuvant chidamide combined with chemotherapy in patients with hormone receptor-positive, human epidermal growth factor receptor 2-negative breast cancer (MUKDEN 05): a multicentre, single-arm, phase 2 trial

**DOI:** 10.1016/j.lanwpc.2025.101700

**Published:** 2025-09-27

**Authors:** Jinqi Xue, Huilian Shan, Fang Qiu, Nan Niu, Guanglei Chen, Qianshi Xu, Xi Gu, Fei Xing, Yongqing Xu, Xinyu Zheng, Guijin He, Hong Xu, Hao Zhang, Dong Song, Ye Han, Meiyue Tang, Shuo Cao, Yang Song, Ran Zheng, Yafei Zhao, Gege Jiao, Mingxin Liu, Caigang Liu

**Affiliations:** a Department of Oncology, Shengjing Hospital of China Medical University, Shenyang, China; b Cancer Stem Cell and Translational Medicine Laboratory, Shengjing Hospital of China Medical University, Shenyang, China; c Innovative Cancer Drug Research and Development Engineering Center of Liaoning Province, Shenyang, China; d Department of Breast Surgery, The First Affiliated Hospital of China Medical University, Shenyang, China; e Department of Breast Surgery, Liaoning Cancer Hospital and Institute, Shenyang, China; f Department of Breast Surgery, The First Affiliated Hospital of Jilin University, Changchun, China

**Keywords:** Chidamide, Chemotherapy, Neoadjuvant therapy, Breast cancer, Chinese

## Abstract

**Background:**

Existing neoadjuvant therapies for hormone receptor-positive, human epidermal growth factor receptor 2-negative (HR+/HER2−) breast cancer have not achieved optimal clinical outcomes, thus prompting the investigation of novel treatment strategies. Given the emerging significance of HDAC inhibitors in the management of advanced breast cancer, this study investigated the efficacy and safety of chidamide plus chemotherapy as neoadjuvant therapy for patients with HR+/HER2− breast cancer.

**Methods:**

This single arm, open-label, multicentre, phase 2 trial was conducted at three hospitals in China. Patients aged 18–75 years who had treatment-naïve, stage II–III HR+/HER2− breast cancer with an Eastern Cooperative Oncology Group performance status of 0–1 were enrolled. Patients received oral chidamide at a dose of 20 mg on days 1, 4, 8, and 11 of each 21-day cycle. The chemotherapy regimen consisted of four cycles of epirubicin (90 mg/m^2^) and cyclophosphamide (600 mg/m^2^) administered every three weeks, followed by four cycles of docetaxel (100 mg/m^2^) administered every three weeks. The primary endpoint was the rate of residual cancer burden (RCB) 0–I. This study is registered with ClinicalTrials.gov, NCT05400993.

**Findings:**

Between May 23, 2022 and July 6, 2023, a total of 54 female patients (median age: 50 years [range, 26–75]) were recruited. The RCB 0–I rate was 35.2% (19 of 54, 95% confidence interval [CI], 22.7%–49.4%). The most frequent grade 3 or 4 adverse events were decreased neutrophil count (70%) and decreased white blood cell (67%). No treatment-related deaths occurred.

**Interpretation:**

The combination of chidamide and chemotherapy shows the potential to be an alternative neoadjuvant therapy option for patients with HR+/HER2− breast cancer.

**Funding:**

Shenyang Municipal Science and Technology Program (Grant No. 22-321-32-18), Shenzhen Chipscreen Biosciences Co., Ltd.

**Research in context**

**Evidence before this study**

We searched PubMed for phase 2 or 3 clinical trials of neoadjuvant therapy in patients with hormone receptor-positive, human epidermal growth factor receptor 2-negative (HR+/HER2−) breast cancer up to March 31, 2025. The search terms used were “hormone receptor-positive”, “HER2-negative”, “breast cancer”, and “neoadjuvant OR preoperative” without language restrictions. The rate of residual cancer burden 0–I was 6.1%–7.7% with cyclin-dependent kinase 4/6 inhibitors plus endocrine therapy, 30.7%–35% with programmed cell death-1 inhibitors plus chemotherapy, 17.6% with trastuzumab deruxtecan, and 6.3% with trastuzumab deruxtecan plus endocrine therapy.

**Added value of this study**

Our study is the first prospective study to report the efficacy and safety of chidamide plus chemotherapy as neoadjuvant therapy in patients with HR+/HER2− breast cancer. The results suggest the potential of this combination therapy to improve residual cancer burden.

**Implications of all the available evidence**

Our results need to be further validated in a randomized controlled trial to fully determine the role of chidamide in this context. Randomization strategy needs discussion to balance ethical concerns and scientific rigor.

## Introduction

Hormone receptor-positive, human epidermal growth factor receptor 2-negative (HR+/HER2−) breast cancer accounts for approximately 70% of breast cancer cases.^1^ Among these, 45%–55% present with locally advanced disease or a high tumor burden, necessitating neoadjuvant therapy to improve surgical outcomes.^2^ Despite the generally indolent biological behavior, HR+/HER2− breast cancer exhibits reduced sensitivity to standard neoadjuvant chemotherapy strategies, resulting in low rates of pCR.^3^ Current neoadjuvant chemotherapy or endocrine therapy for this population yield a limited pathological complete response (pCR) rate (0–10%) and an objective response rate of around 60%.^4^ While improving pCR rate is a potential avenue for enhancing treatment outcomes, it is important to recognize that pCR does not always strongly correlate with event-free survival (EFS) or overall survival (OS) in ER+/HER2−ER2 overall survival (OS) in ER+/HE/HEh event-free survival (EFS) or overall survival (OS) in ER+/HE/HE not always strongly correlate with event-free survival (EFS) or overall survival (OS) in ER+/HE/HEnvestigation of novel treatment strategies. Given ts who are most likely to benefit from escalated approaches.

Given the intrinsic characteristics of HR+/HER2− breast cancer, it is imperative to explore and develop innovative treatment paradigms to improve outcomes. Several clinical trials have begun to explore the efficacy of novel agents, such as cyclin-dependent kinase (CDK) 4/6 inhibitors, immune checkpoint inhibitors (ICIs), and antibody-drug conjugates, in the neoadjuvant setting for HR+/HER2− breast cancer.^3^ Combination therapy with CDK4/6 inhibitors and endocrine therapy, once considered as a breakthrough for HR+/HER2− advanced breast cancer, has shown limited efficacy in the neoadjuvant setting, with a pCR rate of less than 5%.^4^ In contrast, adjuvant therapy with CDK4/6 inhibitors has shown significant clinical benefit in patients with high-risk HR+/HER2− early breast cancer. Studies such as monarchE and NATALEE have confirmed improvement in invasive disease-free survival with the addition of CDK4/6 inhibitors to standard endocrine therapy in selected high-risk population,^5^^,^^6^ leading to regulatory approvals worldwide. The CheckMATE 7FL and KEYNOTE-756 studies demonstrated that adding ICIs to neoadjuvant chemotherapy achieved a pCR rate of about 24% in high-risk, early-stage estrogen receptor-positive (ER+)/HER2− breast cancer.^7^^,^^8^ However, this benefit appears to be concentrated in ER-low tumors, many of which exhibit biological features closer to triple-negative breast cancer. Additionally, mature EFS and OS data from these studies remain lacking. The TALENT study found that neoadjuvant trastuzumab deruxtecan achieved a pCR rate of 6.3% as monotherapy and no pCR when combined with endocrine therapy in HR+/HER2-low breast cancer.^9^ Given the significant heterogeneity of this breast cancer subtype, it's imperative to explore and develop innovative treatment paradigms to improve outcomes.

Dysregulated histone deacetylase (HDAC) expression is prevalent across both solid and hematological malignancies. Chidamide, the first oral subtype-selective HDAC inhibitor, selectively targets HDAC subtypes 1, 2, 3, and 10.^10^ In patients with HR+/HER2− advanced breast cancer, chidamide in combination with endocrine therapy has shown clinical efficacy.^11^ However, in the neoadjuvant setting, chidamide combined with endocrine therapy achieved a pCR rate of only 5%,^12^ underscoring the need for further exploration. Preclinical studies have demonstrated the synergistic efficacy of HDAC inhibitors when combined with chemotherapy.^13^, ^14^, ^15^ Chidamide plus chemotherapy has been explored in advanced solid tumors (including non-small cell lung cancer and triple-negative breast cancer),^16^^,^^17^ providing useful safety data.

Here we conducted this study to evaluate the efficacy and safety of chidamide combined with chemotherapy as neoadjuvant therapy in patients with HR+/HER2− breast cancer.

## Methods

### Study design and participants

MUKDEN 05 was an investigator-initiated, open-label, multicentre, single-arm, phase 2 study conducted at three hospitals in China. Eligible patients were women aged 18–75 years who had histologically confirmed HR+/HER2− invasive breast cancer; had HER2 status of 0 or 1+ by immunohistochemistry (IHC), or 2+ by IHC with negative fluorescence in situ hybridization (FISH) results; had treatment-naïve stage II–III disease; had a Karnofsky performance status score ≥70; and had adequate organ function. Key exclusion criteria included any prior anti-tumor treatment (chemotherapy, radiotherapy, targeted therapy, or endocrine therapy); bilateral, inflammatory, or occult breast cancer; any other malignancies within the past 5 years, except for cured cervical carcinoma in situ; and pregnancy or lactation. Full eligibility criteria are detailed in the study protocol. This study is registered with ClinicalTrials.gov, NCT05400993.

### Procedures

Eligible patients received chidamide plus chemotherapy for eight 21-day cycles. The dosage and administration schedule of chidamide (20 mg twice weekly) were based on previous clinical trials when combined with chemotherapy in solid tumors.^16^^,^^17^ Considering the overlapped hematological toxicities of chidamide and chemotherapy, chidamide was administered on days 1, 4, 8, and 11 of each cycle (i.e., two weeks on and one week off)^17^ to provide a time window for bone marrow recovery. In Accordance with National Comprehensive Cancer Network (NCCN) breast cancer guideline, the chemotherapy regimen consisted of four cycles of epirubicin (90 mg/m^2^) and cyclophosphamide (600 mg/m^2^) administered every three weeks, followed by four cycles of docetaxel (100 mg/m^2^) administered every three weeks. Stepwise dose reductions of chidamide, from 20 mg to 10 mg, were permitted to manage toxicities. Similarly, up to two dose reductions of epirubicin, cyclophosphamide and docetaxel (each by 25%) were permitted. Detailed guidelines for dose interruptions and modifications are described in the study protocol. If patients had disease progression during neoadjuvant therapy, they could either switch to surgery or receive alternative neoadjuvant therapy. Surgery was performed within four weeks after the final dose of chidamide to allow recovery from adverse events. Adjuvant therapies, including radiotherapy (if indicated) and endocrine therapy, were recommended in line with NCCN guidelines.

Breast and axillary lymph node assessments via ultrasound and magnetic resonance imaging were conducted at baseline, after every two cycles of neoadjuvant therapy, and before surgery. Clinical response was locally assessed by two experienced radiologists at each site using magnetic resonance imaging and evaluated according to the Response Evaluation Criteria in Solid Tumors (RECIST) version 1.1. Pathological responses, including residual cancer burden (RCB) and pCR, were determined from surgically resected breast tissues and axillary lymph nodes. Two professional pathologists at each site performed the assessments locally. RCB was classified into four categories: no residual disease, minimal residual disease, moderate residual disease, and extensive residual disease.^18^ The RCB classification (0–III) was determined using the MD Anderson RCB Calculator based on 5 factors: primary tumor bed area, overall cancer cellularity, percentage of carcinoma in situ, positive lymph nodes number, and the largest metastasis diameter.^18^ Ki67 expression was assessed at baseline and after surgery using IHC with a Ki67-specific rabbit monoclonal antibody (Ventana, catalog number H36867).

Adverse events were monitored from the initiation of neoadjuvant therapy until 28 days after the final dose. All adverse events were evaluated using the National Cancer Institute Common Terminology Criteria for Adverse Events version 5.0.

An independent Data and Safety Monitoring Board (DSMB) was not established for this study.

### Study endpoints

The primary endpoint was the proportion of patients achieving RCB 0–I. Patients who did not undergo surgery were considered as not achieving RCB 0–I. Secondary endpoints included pCR (ypT0/is ypN0) rate and breast pCR (bpCR; ypT0/is) rate, objective response rate (defined as the proportion of patients with complete or partial response), breast-conserving surgery rate, and safety.

### Statistical analysis

We adopted Simon's minimax two-stage design to calculate the required sample size. A large-scale pooled analysis showed that the RCB 0 rate of neoadjuvant therapy was 11.1% and the RCB I rate was 10.8% in patients with HR+/HER2− breast cancer.^19^ Based on this pooled analysis, we established the null hypothesis of RCB 0–I rate as 20% and the alternative hypothesis as 35%, with a two-sided significance level of 0.05 and a power of 80%. In the first stage, 31 patients were required; the study would be terminated if no more than six patients achieved RCB 0–I. If this threshold was met, the study would proceed to the second stage, enrolling additional 22 patients.

Efficacy was analyzed separately in the intent-to-treat (ITT) population and the per-protocol (PP) set. The ITT population included all enrolled patients, while the PP set comprised patients who were compliant with the study protocol requirements. The safety analysis set included all patients who received at least one dose of the study drug and had available safety data.

All the statistical analyses were performed with SAS 9.4 (North Carolina, USA). Continuous data were presented as mean (standard deviation), median (range), or mean (95% confidence interval [CI]). Categorical variables were expressed as frequency (percentage). The 95% CIs for the RCB 0–I rate, pCR rate, breast-conserving surgery rate and objective response rate were calculated using the Clopper–Pearson method. The difference in Ki67 expression between baseline and surgical breast tissue samples was analyzed by paired t-test. Statistical significance was defined as a two-sided p-value of <0.05.

### Ethics approval

The trial protocol was approved by the Medical Ethics Committees of Shengjing Hospital of China Medical University (No. 2022PS011T, May 23, 2022), the First Affiliated Hospital of China Medical University (No. [2023]2022-493-2, March 10, 2023), and The First Affiliated Hospital of Jilin University (No. 23K008-001, February 23, 2023). All participating sites complied with the Declaration of Helsinki and Good Clinical Practice guidelines. All patients provided written informed consent prior to enrollment.

An independent Data and Safety Monitoring Board (DSMB) was not established for this investigator-initiated trial, as the study involved approved, commercially available drugs with well-characterized safety profiles. Considering the relatively small sample size, safety monitoring was conducted by the study investigators in close coordination with the institutional ethics committee, which performed regular reviews. This monitoring arrangement was consistent with institutional guidelines and deemed appropriate given the scope of the study.

### Role of the funding source

The funder had no role in study design, data collection, data analysis, data interpretation, or writing of the report.

## Results

**Fig. 1:**
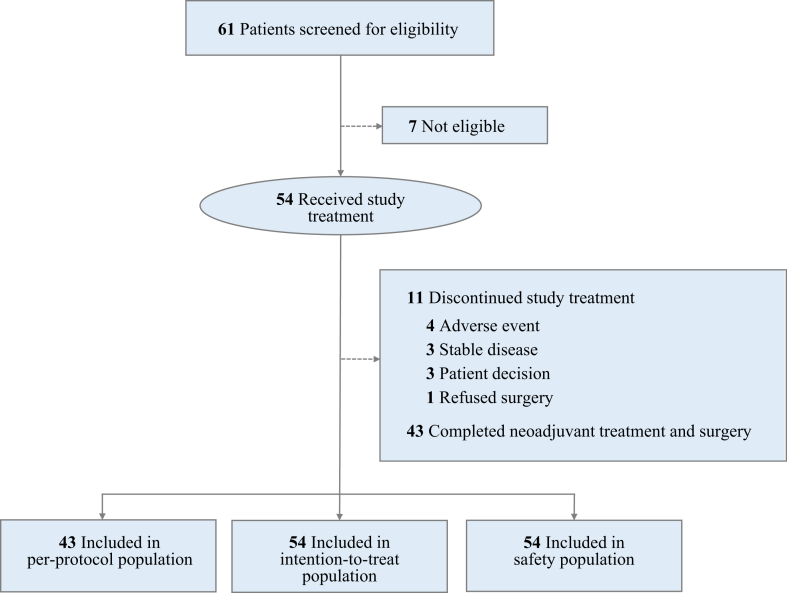
**Trial profile**.

Between May 23, 2022 and July 6, 2023, a total of 61 female patients were screened for eligibility. From May 27, 2022 onward, 54 patients were enrolled and treated (ITT population). Ten patients did not follow the per-protocol neoadjuvant therapy due to adverse events (n = 4), no response (sustained stable disease per RECIST 1.1; n = 3), and patient decision (n = 3). One patient refused surgery. Thus, 43 patients were included in the PP set. A detailed breakdown of these deviations is presented in Fig. 1. Baseline characteristics are described in Table 1. Of 54 patients, 37 (69%) had stage IIB–III disease and 39 (72%) presented with lymph node metastases (N1 or N2). The mean expression of ER and progesterone receptor (PR) in core-needle biopsy tissue samples at baseline were 88% and 65%, respectively. The mean Ki67 expression at baseline was 40%. HER2 status included IHC −/1+ (61%) and IHC 2+/FISH− (39%).

**Table 1:** Baseline characteristics.

	Patients (n = 54)
Age, median (range), years	50 (26–75)
≤50	32 (59%)
>50	22 (41%)
Menopausal status	
Pre- or perimenopausal	34 (63%)
Postmenopausal	20 (37%)
Stage	
IIA	17 (31%)
IIB	22 (41%)
III	15 (28%)
Nodal status	
N0	15 (28%)
N1	21 (39%)
N2	18 (33%)
Tumor size	
T1	7 (13%)
T2	41 (76%)
T3–4	6 (11%)
ER expression, mean (SD), %	88% (13%)
PR expression, mean (SD), %	65% (32%)
Ki67 expression, mean (SD), %	40% (18%)
HER2 status	
IHC −/1+	33 (61%)
IHC 2+/FISH−	21 (39%)

Data are n (%), unless otherwise indicated.

ER, estrogen receptor; SD, standard deviation; PR, progesterone receptor; HER2, human epidermal growth factor receptor 2; IHC, immunohistochemistry; FISH, fluorescence in situ hybridization.

**Fig. 2:**
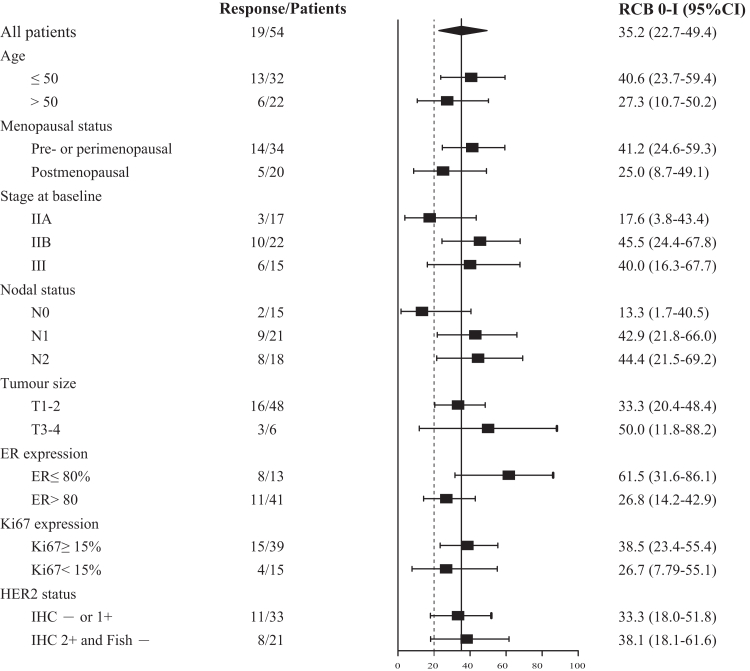
**Exploratory subgroup analyses of RCB 0–I rate (the primary outcome of the study) by different baseline characteristics**. Data are presented as RCB 0–I rate (%) and 95% CI. The dotted line indicates the null hypothesis of 20%. RCB, residual cancer burden; ER, estrogen receptor; HER2, human epidermal growth factor receptor 2; IHC, immunohistochemistry; FISH, fluorescence in situ hybridization; CI, confidence interval.

In line with the study protocol, 31 patients were recruited during the first stage. After receiving the neoadjuvant therapy, 13 patients (41.9%, 95% CI, 24.5%–60.9%) achieved RCB 0–I, meeting the minimum threshold of responders required to proceed with the study. In the overall ITT population, the primary endpoint was achieved, with 19 patients (35.2%, 95% CI, 22.7%–49.4%) of 54 patients achieving RCB 0–I. The pCR rate was 9.3% (5/54, 95% CI, 3.1%–20.3%), and the bpCR rate was 20.4% (11/54, 95% CI, 10.6%–33.5%) in the ITT population. In the PP set, the RCB 0–I rate was 41.9% (18/43, 95% CI, 27.0%–57.9%), with a pCR rate of 11.6% (5/43, 95% CI, 3.9%–25.1%) and a bpCR rate of 25.6% (11/43, 95% CI, 13.5%–41.2%; Table 2). A post-hoc exploratory analysis of the primary endpoint (RCB 0–I) by baseline characteristics did not reveal any subgroup with substantial benefit from chidamide plus chemotherapy, possibly due to the small sample size (Fig. 2). This study did not observe a correlation between HER2 expression status and pathological response (Appendix Fig. S1). Objective response was observed in 42 patients (77.8%, 95% CI, 64.4%–88.0%) in the ITT population and in 37 patients (86.0%, 95% CI, 72.1%–94.7%) in the PP set (Table 2).

**Table 2:** Pathological and clinical responses.

	Intention-to-treat population (n = 54)	Per-protocol set (n = 43)
Residual cancer burden score		
0	5 (9%)	5 (12%)
I	14 (26%)	13 (30%)
II	22 (41%)	15 (35%)
III	13 (24%)	10 (23%)
Total pathological complete response	5 (9%)	5 (12%)
Pathological complete response in breast	11 (20%)	11 (26%)
Best overall response		
Complete response	4 (7%)	4 (9%)
Partial response	38 (70%)	33 (77%)
Stable disease	12 (22%)	6 (14%)
Objective response	42 (78%)	37 (86%)

Data are n (%).

After neoadjuvant therapy, the proportion of patients with opportunity for breast-conserving surgery increased from 5.6% (3/54) to 42.6% (23/54). However, 13 patients opted not to undergo breast-conserving surgery. Consequently, 10 patients (18.5%, 95% CI, 9.3%–31.4%) in the ITT population and 9 patients (20.9%, 95% CI, 10.0%–36.0%) in the PP set underwent breast-conserving surgery.

**Fig. 3:**
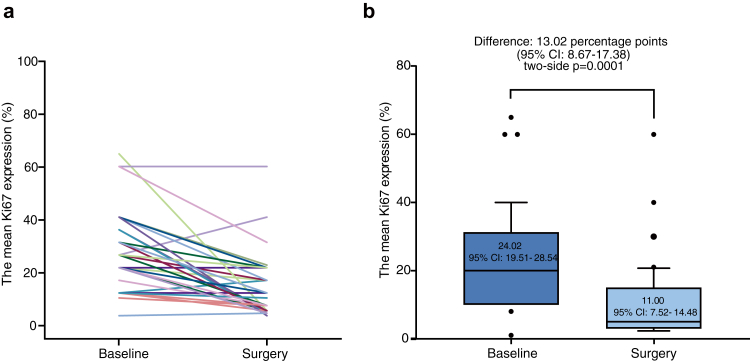
**Ki67 expression at baseline and surgery**. (a) Individual data (n = 42). (b) Mean expression of Ki67 (n = 42). The Ki67 expression in 12 of 54 patients were not evaluated due to the lack of detectable tumor cells in the surgical samples (n = 11) or no surgery (n = 1). CI, confidence interval.

Exploratory analyses of Ki67 expression were conducted at baseline and surgery. Ki67 data were unavailable in 12 patients due to the absence of detectable tumor cells in surgical samples (n = 11) or the patient's decision to decline surgery (n = 1). Among the 42 paired baseline biopsy and surgical breast tumor samples analyzed, the mean Ki67 expression decreased from 24.02% (95% CI, 19.51%–28.54%) at baseline to 11.00% (95% CI, 7.52%–14.48%) in surgical samples after neoadjuvant therapy (p < 0.001; Fig. 3).

In the safety analysis set, all 54 patients experienced at least one adverse event. The most common adverse events were alopecia (50 [93%]), nausea (48 [89%]), anorexia (47 [87%]), decreased white blood cell (47 [87%]), decreased neutrophil count (46 [85%]), fatigue (43 [80%]), vomiting (42 [78%]), and anemia (42 [78%]; Table 3). A total of 44 patients (81%) experienced grade 3 or 4 adverse events, with the most common being decreased neutrophil count (38 [70%]), decreased white blood cell (36 [67%]), and anemia (3 [6%]). Dose interruptions for chidamide were required in 38 patients (70%). Four patients (7%) required dose reduction of chidamide to 10 mg, attributed to grade 4 decreased neutrophil count (n = 3) or grade 4 decreased platelet count (n = 1). Additionally, four patients (7%) discontinued the study treatment due to adverse events. No treatment-related deaths occurred in this study.

**Table 3:** Treatment-emergent adverse events.

	Patients (n = 54)		
	Grade 1 or 2	Grade 3	Grade 4
Alopecia	50 (93%)	–	–
Nausea	48 (89%)	0	0
Anorexia	47 (87%)	0	0
White blood cell decreased	11 (20%)	21 (39%)	15 (28%)
Neutrophil count decreased	8 (15%)	6 (11%)	32 (59%)
Fatigue	42 (78%)	1 (2%)	0
Vomiting	42 (78%)	0	0
Anemia	39 (72%)	3 (6%)	0
Alanine aminotransferase increased	34 (63%)	1 (2%)	0
Aspartate aminotransferase increased	31 (57%)	0	0
Diarrhea	28 (52%)	1 (2%)	0
Platelet count decreased	27 (50%)	0	1 (2%)
Abdominal pain	24 (44%)	0	0
Triglyceride increased	23 (43%)	0	0
Dysgeusia	21 (39%)	0	0
Cholesterin increased	20 (37%)	0	0
Gastrectasia	19 (35%)	0	0
γ-glutamyltransferase increased	18 (33%)	1 (2%)	0
Hypoproteinemia	18 (33%)	0	0
Constipation	16 (30%)	0	0
Stomachache	15 (28%)	0	0
Fever	13 (24%)	0	0
Insomnia	10 (19%)	0	0
Palpitation	9 (17%)	0	0
Dizziness	7 (13%)	0	0
Oral ulcer	7 (13%)	0	0

Data are n (%).

## Discussion

The MUKDEN 05 study represents a pioneering clinical investigation into the efficacy and safety of neoadjuvant chidamide combined with chemotherapy for HR+/HER2− breast cancer. The study has successfully met its primary endpoint, achieving an RCB 0–I rate of 35.2% and demonstrating a manageable safety profile with GCSF prophylaxis. These findings suggest that this combination therapy has the potential to become a promising neoadjuvant strategy for patients with HR+/HER2− breast cancer.

HR+/HER2− breast cancer is well recognized as a clinically heterogenous subtype of breast cancer, which exhibits variability in ER and PR levels, histology, and Ki67 expression.^20^ Comprehensive analyses and subtyping—such as PAM50 subtype^1^ and SNF subtype^21^—have further elucidated the biological complexity of HR+/HER2− breast cancer. This heterogeneity underpins significant differences in patient prognosis and treatment response, underscoring the critical importance of personalized therapeutic strategies.

The treatment landscape for HR+ breast cancer is rapidly evolving, driven by the introduction of novel agents and the increasing application of gene expression profiling to guide treatment selection. The neoadjuvant approach for early-stage, HR+/HER2− breast cancer provides an excellent opportunity to analyze both the clinical and biological heterogeneity of this subtype and to refine personalized therapeutic strategies. Clinical trials have reported a pCR rate of approximately 10% following neoadjuvant chemotherapy or endocrine therapy in HR+/HER2− breast cancers.^4^ The combination of CDK4/6 inhibitors and aromatase inhibitors in neoadjuvant therapy has been hypothesized to enhance therapeutic efficacy. However, neither the PALLET nor neoMONARCH studies demonstrated an increase in pCR rate (3.3%–4%).^22^^,^^23^ Given the relatively low pCR rate achieved with neoadjuvant therapy in HR+/HER2− breast cancer and the fact that pCR is not a reliable predictor of survival,^24^ RCB 0–I rate has been adopted as the primary endpoint in some neoadjuvant studies for HR+/HER2− breast cancer. Studies have shown that RCB 0–I can better predict survival.^19^ However, the CORALLEEN and NeoPAL studies failed to show notable improvement in RCB 0–I rate (6.1%–7.7%).^25^^,^^26^ Recently, neoadjuvant ICIs combined with chemotherapy were explored in HR+/HER2− breast cancer. In the CheckMate 7FL study, pCR rate was 24.5% with nivolumab group compared to 13.8% in the placebo group, and the RCB 0–I rate was 30.7% and 21.3%, respectively.^7^ The KEYNOTE-756 study reported a pCR rate of 24.3% in the pembrolizumab group versus 15.6% in the placebo group, and the RCB 0–I rate was 35% and 23.6%, respectively.^8^ As an emerging anti-HER2 antibody-drug conjugate, neoadjuvant trastuzumab deruxtecan showed an RCB 0–I rate of 17.6% in the monotherapy group and 6.3% when combined with aromatase inhibitors for patients with HR+/HER2-low breast cancer.^9^ The biological heterogeneity in HR+/HER2− breast cancer highlight an enduring need for innovative therapeutic approaches to improve treatment efficacy.

Chidamide, recognized as a subtype-selective HDAC inhibitor, can alter the epigenetic landscape of tumor cells to enhance their susceptibility to chemotherapeutic agents and modulate genes or proteins implicated in drug resistance pathways, thereby reducing the likelihood of chemoresistance in tumor cells.^27^^,^^28^ In the MUKDEN 05 study, the RCB 0–I rate was 35.2% with chidamide plus chemotherapy, higher than that with chemotherapy alone (11.8%–21.3%)^7^^,^^8^^,^^25^^,^^26^ and trastuzumab deruxtecan with or without aromatase inhibitors (6.3%–17.6%),^9^ and similar to that with ICIs plus chemotherapy (30.7% in the CheckMate 7FL study and 35% in the KEYNOTE 756 study).^7^^,^^8^ Our safety data suggest that the most common adverse events with chidamide plus chemotherapy were hematological toxicities (e.g., thrombocytopenia and neutropenia) and mild gastrointestinal reactions, without concerns about immune-related adverse events (such as pneumonitis, colitis, and endocrinopathies). Importantly, the side effects of chidamide were generally manageable and reversible, whereas immune-related adverse events from ICIs may require prolonged immunosuppressive management. Thus, chidamide may be an alternative option for patients who are ineligible for or intolerant to immunotherapy. Notably, in the MUKDEN 05 study, the objective response rate reached 77.8%, which is clinically meaningful for high-risk ER+/HER2+ breast cancer patients with urgent need of downstaging for surgery. However, the pCR rate in our study was still low (9%) and the bpCR rate was 20%, showing no improvement. Notably, in the KEYNOTE-756 study, the pCR rate of Chinese subgroup receiving pembrolizumab combined with chemotherapy was only 12.5%.^8^ The underlying reasons warrant further researches.

In the field of neoadjuvant therapy, Ki67 emerges as a pivotal biomarker for monitoring treatment efficacy. Neoadjuvant endocrine therapy alone has been shown to significantly reduce Ki67 expression in ER+/HER2− breast cancer, serving as a key marker of long-term outcomes and facilitating the refinement of treatment strategies.^29^ Notably, Ki67 expression in our study significantly decreased from 24.0% at baseline to 11.0% after neoadjuvant therapy. This change reflects the anti-proliferative effect of chidamide plus chemotherapy and may also predict prognosis, which warrants further investigation.

The adverse event profile of chidamide is predominantly characterized by hematological toxicities, especially for neutropenia.^30^^,^^31^ Fatigue is also a commonly documented adverse event in patients receiving chidamide.^30^^,^^31^ Grade 3 or 4 adverse events with chidamide plus chemotherapy were mainly hematological toxicities, consistent with previous studies of chemotherapy.^32^ Considering the overlapped hematological toxicities, chidamide was administered twice weekly for two weeks followed by one-week break in each cycle, and epirubicin cyclophosphamide (EC) were administered every three weeks rather than every two weeks. Even under this circumstance, this combination regimen resulted in an unexpectedly high rate of grade 4 neutropenia (59%). Routine use of granulocyte-colony stimulating factor (G-CSF) was not mandated in this study, which should be essential in future study. In addition, the feasibility of chidamide plus accelerated EC regimen with standard G-CSF support also need further exploration.

The study has some limitations. First, this was a single-arm study. The lack of a control group and the relatively small sample size might potentially impact the reliability of efficacy outcomes, and the contribution of chidamide could not be fully determined. Second, this study only enrolled Chinese patients. Considering the regional population differences and variations in health care standards, our results need to be validated in other populations. Third, 3-weekly EC regimen was used instead of the accelerated EC regimen, which might lead to diminished efficacy. Fourth, EFS and OS were not included as secondary endpoints, and long-term survival outcomes of chidamide plus chemotherapy are needed. Finally, the absence of correlative biomarker analyses limits insights into the biological mechanisms underlying the treatment efficacy. Addressing these limitations in future randomized controlled trials will be critical for refining the role of chidamide-based neoadjuvant therapy in patients with ER+/HER2− breast cancer. From a policy and implementation perspective, alternative randomization strategies should be explored to balance ethical concerns and scientific rigor. As proposed by Venkataramani et al.,^33^ randomization at the program or institutional level rather than the individual patient level may be a more acceptable approach when evaluating interventions that are already used in clinical practice. This framework can facilitate evidence-based policy decisions while ensuring equitable access to promising therapies.

In conclusion, the study pioneers the investigation of chidamide combined with chemotherapy as neoadjuvant therapy for HR+/HER2− breast cancer. This regimen shows the potential to improve RCB with a manageable safety profile, offering a promising alternative treatment option for these patients. Further large-scale validation of these findings is necessary to solidify its place in clinical practice.

## Contributors

CL conceived and designed the study. JX, HS, FQ, NN, GC, QX, XG, FX, YX, XZ, GH, HX, HZ, DS, YH, MT, SC, YS, RZ, YZ, and ML recruited patients. JX, NN, RZ, ML, and YZ collected the data. CL and JX had accessed and verified the data. NN, RZ, and ML contributed to the analysis and interpretation of data. NN drafted the manuscript. All authors contributed to critical review of the manuscript. CL contributed to the study supervision. CL contributed to the administrative support. CL was responsible for the decision to submit the manuscript.

## Data sharing statement

The datasets generated and/or analyzed during the study are available from the corresponding author Caigang Liu on reasonable request; de-identified clinical data and experimental data will be shared and transferred to the inquiring investigator after the approval of the institutional ethics committee.

## Declaration of interests

All authors declare no conflicts of interest.

## Appendix A Supplementary data

Supplementary Materials

Protocol
