# Supplementary material for: Neoadjuvant chidamide combined with chemotherapy in patients with hormone receptor-positive, human epidermal growth factor receptor 2-negative breast cancer (MUKDEN 05): a multicentre, single-arm, phase 2 trial
Source: Lancet Reg Health West Pac. 2025 Sep 27;63:101700. doi: 10.1016/j.lanwpc.2025.101700 (PMC12510053; doi:10.1016/j.lanwpc.2025.101700)
Supplement: Supplementary Materials [file mmc1.pdf]

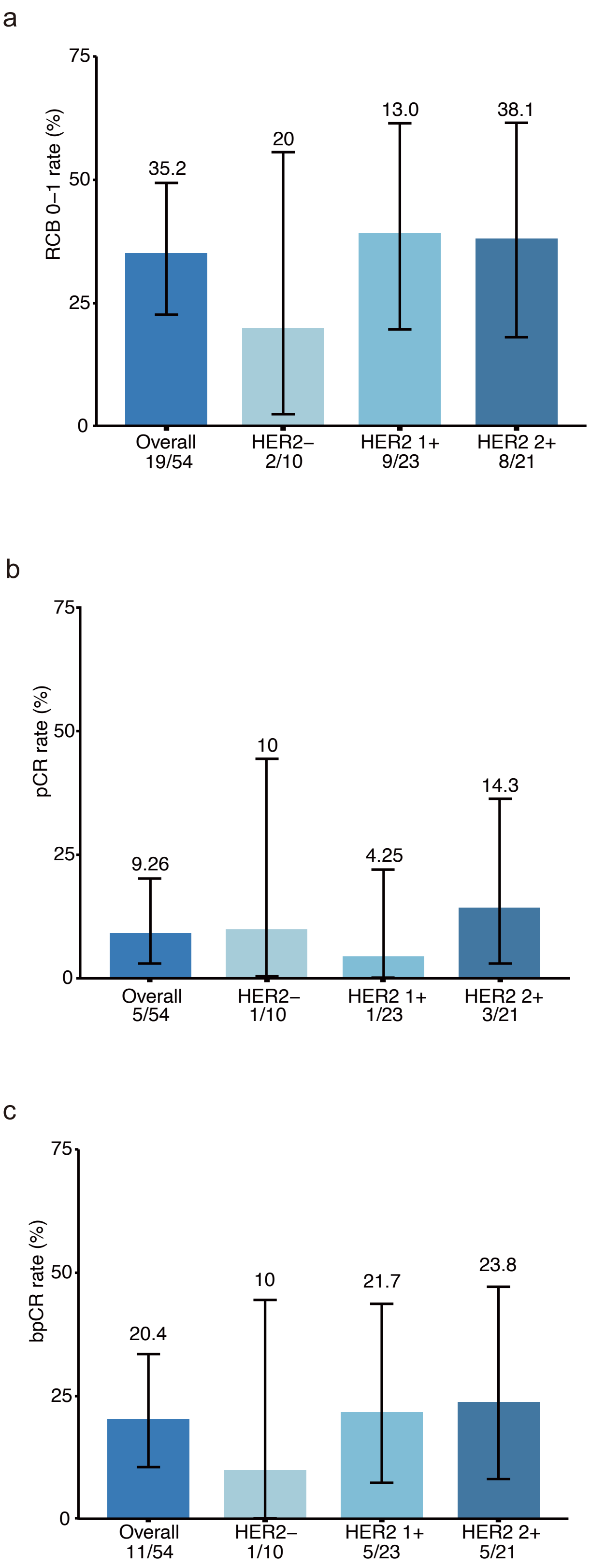

Fig. S1: Pathological response analyses by HER2 expression. (a) RCB 0-I rate. (b) pCR rate. (c) bpCR rate. HER2, human epidermal growth factor receptor 2; RCB, residual cancer burden; pCR, pathological complete response; bpCR, breast pathological complete response.

A Multicenter, Single-Arm, Prospective, Phase II Clinical Study of Chidamide Combined with Chemotherapy as Neoadjuvant Treatment in HR-Positive/HER2-Negative Breast Cancer

Protocol Version:1.0

Version Date: Apr.15, 2022

Protocol Number: MUKDEN 05

Leading Clinical Study Site

Information of Leading Site

Name：Shengjing Hospital of China Medical University

Address: No. 39, Huaxiang Road, Tiexi District, Shenyang, Liaoning, China

Principal Investigator：Caigang Liu

Signature Page

Sponsor's Signature

I will carefully perform the duties as an investigator in accordance with the Chinese GCP, and personally participate in or directly lead this clinical study. I have read and confirmed this protocol (version no.: 1.0, version date: Apr. 15, 2022). I agree to fulfill my duties in accordance with the laws of China, the Declaration of Helsinki, GCP, and this study protocol, and can only implement them after being approved by the Ethics Committee, unless measures must be taken to protect the safety, rights, and interests of subjects.

Sponsor: Shengjing Hospital of China Medical University

**Caigang Liu**		**15-Apr-2022**
**Principal Investigator (Print)**	**Principal Investigator (Signature)**	**Date of Signature**

Table of Contents

Protocol Abstract . . . 1

List of Abbreviations . . . 16

1. BACKGROUND . . . 19

2 STUDY OBJECTIVES AND ENDPOINTS . . . 21

3 STUDY DESIGN . . . 21

4. Selection and withdrawal of subjects . . . 22

4.1 Inclusion Criteria. . . . 22

4.2 Exclusion Criteria . . . 23

4.3 Withdrawal Criteria. . . . 24

4.3.1 Criteria for Subject Withdrawal . . . 24

4.3.2 Handling of withdrawn subjects . . . 25

5 STUDY DRUGS. . . . 25

6. Concomitant medications . . . 34

7. Study Procedures . . . 35

7.1 Screening Period . . . 36

Tumor hormone receptor status,. . . . 37

7.2 Treatment Period. . . . 38

7.3 Patient Perioperative . . . 39

7.4 End of Study Treatment/Withdrawal from Study . . . 39

7.5 Efficacy Evaluation. . . . 40

7.5.1 Imaging assessment . . . 40

7.5.2 Primary endpoints . . . 41

7.5.3 Secondary endpoints . . . 41

Efficacy endpoints:. . . . 41

8 SAFETY EVALUATION . . . 41

8.1 Physical Examination and Vital Signs . . . 42

8.2 Laboratory Tests . . . 42
Table5 . Laboratory test requirements. . . . 43
8.3 Electrocardiogram . . . 44
8.4 Echocardiography . . . 44
8.5 Adverse Events (AE). . . . 45
8.5.1 Definition of Adverse Events . . . 45
8.5.2 Criteria for the severity of adverse events . . . 45
8.5.3 Causality assessment criteria . . . 46
Table 6. Causality assessment criteria. . . . 46
8.5.4 Recording and reporting of AEs . . . 47
8.6 Serious Adverse Event (SAE) . . . 48
8.6.1 Definition of serious adverse event . . . 48
8.6.2 Potential drug-induced liver injury . . . 49
Table 7. Evaluation criteria for potential drug-induced liver injury . . . 49
8.6.3 Progressive disease . . . 50
8.6.4 Hospitalization. . . . 50
8.6.5 Reporting system of serious adverse events . . . 51
Table 8. SAE reporting method . . . 52
8.6.6 Pregnancy . . . 52
9 STUDY MANAGEMENT . . . 53
9.1 Ethics and Informed Consent . . . 53
9.1.1 Ethics . . . 53
9.1.2 Informed consent . . . 53
9.2 Protocol Amendments . . . 53
9.3 Data Management . . . 54
9.3.1 Data Collection . . . 54
9.3.2 Data management and quality control . . . 54
9.3.3 Data review and monitoring of the study institution . . . 54

9.4 Protocol Violations 55
10 DATA ANALYSIS AND STATISTICAL METHODS 55
10.1 Sample Size Calculation 56
10.2 Statistical Analysis Plan 56
10.2.1 Statistical analysis datasets 56
10.2.2 Statistical analysis methods 56
10.2.3 Statistical software. 57
10.3 Dropouts 57
Appendix I: Clinical Staging Criteria for Breast Cancer (AJCC 8th Edition, TNM Staging of Breast Cancer) 59
Appendix II: Karnofsky Performance Status Score (KPS) 60
Appendix III: 2018 ASCO/CAP Guidelines for HER2 Detection in Breast Cancer 61
Appendix IV: Response Evaluation Criteria in Solid Tumors. 63

Protocol Abstract

**Study Title**	A Multicenter, Single-Arm, Prospective, Phase II Clinical Study of Chidamide Combined with Chemotherapy as Neoadjuvant Therapy inHR+/HER2- Breast Cancer
**Sponsor**	Shengjing Hospital of China Medical University, Liaoning Cancer Hospital & Institute, The First Affiliated Hospital of China Medical University, Bethune First Hospital of Jilin University, Harbin Medical University Cancer Hospital, Dalian University Zhongshan Hospital, Medical science and technology information center of Liaoning Health Industry Group Co., LTD
**Principal Investigator**	Caigang Liu, Hong Xu, Xinyu Zheng, Dong Song, Zhigao li, Honglu Li, and Dianlong Zhang
**Version Date**	Apr. 15,2022
**Version No.**	1.0
**STUDY DRUGS**	Chidamide<br>Epirubicin<br>Cyclophosphamide<br>Docetaxel
	Breast cancer is the most prevalent malignant tumor in women, accounting for 24.2% of all malignant tumors. In 2018, 2.09 million people were diagnosed with breast cancer worldwide, and about 63 million people died of breast cancer. The prevalence of breast cancer in China accounts for 17.1% of all malignant tumors in women. In 2015, there were about 304,000 cases and 70,000 deaths**.** Approximately 75% of breast cancer patients are hormone receptor (Hormone receptor, HR) positive. Neoadjuvant therapy is an important treatment for stage II-III breast cancer. It can reduce the tumor stage and improve the chance of surgery and breast conservation. Clinical studies have shown that patients with early breast

**Study Rationale**	cancer have similar prognosis after neoadjuvant therapy and postoperative adjuvant therapy. In addition, neoadjuvant therapy can also early determine the drug sensitivity and drug resistance in vivo, providing an excellent biological model for efficacy evaluation in the adjuvant treatment of breast cancer.<br><br>HR+/HER2- neoadjuvant therapy for breast cancer is mainly based on anthracycline combined with taxane chemotherapy. Active neoadjuvant chemotherapy can only achieve pCR in about 10% of patients with HR-positive breast cancer. The emergence of new targeted drugs has brought new vitality and hope to patients with HR-positive breast cancer. neoMONARCH, PALLET study, etc. compared CDK4/6 inhibitors in combination with aromatase inhibition<br><br>The efficacy of dose (AI) and single-agent endocrine therapy showed that the combination with CDK4/6 inhibitors could significantly reduce the low tumor cell proliferation index Ki67 and apoptotic index c-PARP led to cellcycle arrest in more patients, but the pathological complete response(pCR) was not improved, and the residual tumor burden (RCB)0/1 was not significantly improved. The LORELEI study explored the efficacy of neoadjuvant treatment with tislelizumab in combination with letrozole, and found that tislelizumab improved the ORR in the overall population, especiallyin the PIK3CA mutation population, but the pCR rate was not significantly different between the overall population and the PIK3CA mutation population. Neoadjuvant endocrine therapy still requires continuous exploration, especially when targeted drugs are rapidly updated and iterated, and combined targeted therapy has become a new research direction to further improve the efficacy of neoadjuvant therapy.<br><br>Basic research shows that abnormal status of epigenetics is related to drug resistance, metastasis, and recurrence of tumors. Histone deacetylase (HDAC) is an important regulator of epigenetic regulation, and targeting HDAC drugs provides a novel strategy for tumor treatment. Chidamide is an oral subtype-selective HDAC inhibitor independently developed in China. It is a novel epigenetic drug belonging to Class 1.1 new drugs, and is the first approved by the China Food and Drug Administration (CFDA) in China for the treatment of relapsed or refractory peripheralT -cell lymphoma (PTCL). The mechanism of

	action of Chidamide: mainly targets the 1,2,3 subtypes in the I classHDAC and the 0 subtype in the IIb class. It not only regulates abnormal epigenetic functions of tumors and induces and activates novel cellular immune functions, but also overcomes chemotherapy resistance and epithelial-mesenchymal transdifferentiation (EMT). In addition, chidamide's unique multiple mechanisms of action can improve the tumor microenvironment, thereby bringing long-term survival benefits to patients. The ACE study evaluated the clinical efficacy and safety of chidamide in combination with exemestane in patients with HR -positive HER2 -negative advanced breast cancer. This is also the first large-scale clinical study of epigenetic modulators in the treatment of solid tumors. The study enrolled postmenopausal patients with HR positive/HER2 -negative advanced breast cancer resistant to endocrine therapy. All enrolled patients were randomly assigned in a 2:1 ratio to receive chidamide with exemestane (n=244) or placebo with exemestane (n=121). Results: The median PFS of patients in Chidamide group was7.4 months vs. 3.8 months in the placebo group (investigator-assessed); median PFS for patients in the chidamide group 9.2 months in the placebo group and 3.8 months in the placebo group (IRC-assessed). ACE Research results suggested that for patients with HR positive HER2 negative advanced breast cancer who have relapsed or progressed after endocrine therapy, the selective HDAC inhibitor chidamide combined with endocrine therapy can significantly improve their survival benefits, providing a new treatment option for these patients. Based on good accessibility and ACE research according to the 2020 and 2021 CSCO clinical guidelines for the diagnosis and treatment of breast cancer, chidamide in combination with Exemestane listed as Class 1A evidence of HR positive advanced breast cancer treatment after failure of tamoxifen/toremifene treatment and failure of non-steroidal AI treatment, Level I recommendation.<br><br>As an epigenetic modulator, chidamide has been shown in vitro and in vivo to induce apoptosis and growth arrest in cancer cells, reverse epithelial-mesenchymal transition and drug resistance in cancer cells, and enhance natural killer cells and antigen-specific CD8+ T lymphocyte-mediated anti-tumor activity. Chidamide was approved in China for the treatment of relapsed or refractory PTCL

	based on the results of a multicenter phase II trial. Real-world studies (RWS) of chidamide in a large patient population further demonstrated good efficacy and potential survival benefits of chidamide in combination with chemotherapy in a relapsed or refractory setting, with the main toxicity associated with chidamide treatment being an acceptable hematologic adverse event. To evaluate the efficacy and safety of chidamide in combination with cyclophosphamide, doxorubicin, vincristine, etoposide, and prednisone (Chi-CHOEP regimen) in the treatment of untreated peripheral T-cell lymphoma (PTCL) in a prospective, multi-center clinical trial. The safe and tolerated dose of Chidamide is 20 mg twice a week. The case fatality rate was 60.2%, and the complete response rate was 40.7%. With a median follow-up of 36 months, the median PFS was 10.7 months, and the 1-, 2-, and 3-year PFS rates were 49.9%, 38.0%, and 32.8%, respectively. The Chi-CHOEP regimen was well tolerated, with approximately two-thirds of patients experiencing grade 3/4 neutropenia. No unexpected adverse events (AEs) were reported and the observed adverse events were manageable.<br>In summary, we envision that neoadjuvant treatment with chidamide combined with chemotherapy will provide a better strategy for patients with HR+/HER2- breast cancer.
**STUDY OBJECTIVES**	To explore the efficacy and safety of chidamide combined with chemotherapy as neoadjuvant therapy in patients with HR+/HER2− breast cancer.
	**Primary endpoints:**<br>RCB 0-1 ratio,<br>**Secondary endpoints:**<br>pCR (ypT0/is, ypN0),<br>bpCR (ypT0/is);<br>Objective response rate (ORR);

**Endpoints**	Breast conserving rate;<br>**Safety endpoints:**<br>Adverse events (AEs) and serious adverse events (SAEs), refer to NCI-CTCAE 5.0.
**Study Design**	This study adopts a multi-center, single-arm, prospective design, and patients with stage II-III HR+/HER2- breast cancer were planned to be enrolled. Patients who received chidamide combined with neoadjuvant chemotherapy. The primary objective was to observe the efficacy and safety of chidamide combined with chemotherapy as neoadjuvant treatment in patients with stage II-III HR+/HER2- breast cancer. Breast MRI and other imaging examinations will be repeated every 2 cycles to evaluate the efficacy. If the efficacy is confirmed, surgery will be performed within 4 weeks after the end of neoadjuvant therapy with chidamide combined with chemotherapy. If the efficacy is SD+/PD, the subject should withdraw from the study. If the toxicity is intolerable, the subject withdraws the informed consent or the investigator judges that the drug must be discontinued. The imaging evaluation will be performed according to RECIST 1.1 criteria, and the evaluation results obtained by the study site are the final results. Within4weeks after surgical treatment, histopathological examination will be performed on the excised tumor tissues and lymph nodes (including the pathology of tumor margins); the pathological sections will be uniformly sent to the leading site for unified review.<br>**Efficacy follow-up**: All subjects must be followed up until tumor progression, death, or withdrawal of informed consent, whichever occurs first.<br>**Safety follow-up**: Follow-up is required until the subject starts to receive other anti-tumor treatments; all AEs return to Grade 0-1 or baseline level or death, whichever comes first.<br>**Survival follow-up**: All subjects will be followed up for survival until death, withdrawal of informed consent, or end of the trial, whichever occurs first.

<table>
<tr><td>Inclusion Criteria</td><td>Subjects must meet all of the following inclusion criteria to be enrolled in this study:
1. Female patients aged ≥ 18 and ≤ 75 years
2. All patients had histopathologically confirmed estrogen receptor (ER) positivity (>10%) HER2 receptor negative. Follow the 2018 ASCO-CAP HER2 Negative Interpretation Guideline criteria;
3. Previously untreated patients with stage II-III tumors meeting the criteria of AJCC 8th edition;
4. KPS score ≥ 70;
5. The functional level of organs must meet the following requirements:
(1) Bone Marrow Function
✓ ANC ≥ 1.5 × 10^9/L (no growth factors used within 14 days);
✓ PLT ≥ 100 × 10^9/L (without corrective treatment within 7 days);
✓ Hb ≥ 100 g/L (without corrective treatment within 7 days);
(2) Liver and kidney function
✓ TBIL ≤ 1.5 × ULN;
✓ ALT and AST ≤ 3 × ULN;
✓ BUN and Cr ≤ 1.5 × ULN and creatinine clearance ≥ 50 mL/min (Cockcroft-Gault formula);
6. Able to undergo needle biopsy;
7. Voluntarily participate in this study, sign the informed consent form, have good compliance, and be willing to cooperate with follow-up visits.</td></tr>
<tr><td></td><td>Subjects with any of the following conditions are not eligible to participate in this clinical study:
1. Previously received other anti-tumor treatments of any form (chemotherapy, radiotherapy, molecular targeted therapy, endocrine therapy, etc.);
2. Concurrent use of any other anti-tumor treatment;</td></tr>
</table>

**Exclusion Criteria**	3. Bilateral breast cancer, inflammatory breast cancer, or occult breast cancer;<br>4. 4. Stage IV breast cancer;<br>5. Breast cancer without histopathological diagnosis;<br>6. Patients with other malignancies within the past 5 years, except for cured cervical carcinoma in situ;<br>7. Patients with severe heart, liver, kidney and other important organ dysfunction;<br>8. Inability to swallow, chronic diarrhea, and intestinal obstruction, with multiple factors affecting drug intake and absorption;<br>9. Participated in other drug clinical trials within 4 weeks prior to enrollment;<br>10. Subjects with a known history of allergy to the drug components of this protocol; a history of immunodeficiency, including positive HIV test, HCV, active viral hepatitis B or other acquired or congenital immunodeficiency diseases, or a history of organ transplantation;<br>11. Any history of heart disease, including: (1) arrhythmia requiring medication or with clinical significance; (2) myocardial infarction; (3) cardiac failure; (4) any other heart disease judged by the investigator to be unsuitable for participating in this trial;<br>12. Pregnant or lactating women, women of childbearing potential with positive baseline pregnancy test results, or women of childbearing potential who are unwilling to take effective contraceptive measures throughout the trial;<br>13. Presence of accompanying diseases that may pose serious risks to the safety of the patient or may affect the patient's ability to complete the study as judged

	by the investigator (including but not limited to severe hypertension, severe diabetes, and active infection that cannot be controlled by drugs);<br>14. History of clear neurological or mental disorders, including epilepsy or dementia. Any other conditions that the investigator considers the patient unsuitable for participating in this study.
**Study Methods**	Patients with stage II-III HR+/HER2- breast cancer who meet the inclusion criteria will be enrolled from the start date of the study.<br>Chidamide: 20 mg, po, bid, po, 2 weeks on, 1 week off<br>Epirubicin 90 mg/m^2, ivgtt, d1<br>Cyclophosphamide 600 mg/m^2, ivgtt, d1<br>Q3w, 4 cycles<br>Sequential<br>Chidamide:20 mg, po, bid, po, 2 weeks on, 1 week off<br>Docetaxel 100 mg/m^2, ivgtt, d1<br>Q3w,4 cycle<br>Breast MRI and other imaging examinations will be repeated every 2 cycles to evaluate the efficacy. If the efficacy is confirmed, surgery will be performed within 4 weeks after the end of neoadjuvant therapy in cycle 8. If the efficacy is SD+/PD, the subject should withdraw from the study. If the toxicity is intolerable, the subject withdraws the informed consent or the investigator judges that the drug must be discontinued. The imaging evaluation will be performed according to RECIST 1.1 criteria, and the evaluation results obtained by the study site are the final results. Within 4 weeks after surgical treatment, histopathological examination will be performed on the excised tumor tissues and lymph nodes (including the pathology of tumor margins); the pathological sections will be uniformly sent to the leading site for unified review.

**Study Progress**	Anticipated enrollment of the first subject: May, 2022<br><br>Anticipated enrollment of the last subject: May, 2023

	**Screening period**	**Neoadjuvant therapy**			**Surgery**	**End of Study/Withdrawal**	**Safety Follow-up** [22]
	D−14 to D−1	C1–C8			Within 4 weeks (> 2 weeks) after C8		
		D7 ± 3	D14 ± 3	D21 ± 3			
Signing of informed consent form	X						
Demographics [1]	X						
Medical History [2]	X						
Concomitant medications [3]	X	X					
Physical Examination [4]	X				X (before surgery)	X	
Vital Signs [5]	X			X	X (before surgery)	X	
KPS score	X			X	X (before surgery)	X	
Adverse Events [6]	From signing the informed consent form to 21 days after the last dose						
Routine blood test[7]	X	X	X	X	X (before surgery)	X	
Blood biochemistry test [8]	X			X	X (before surgery)	X	
Coagulation function test[9]	X			X	X (before surgery)		
Urinalysis[10]	X			X	X (before surgery)	X	

Routine stool test[11]	X			X	X (before surgery)	X	
Infectious Disease Screening [12]	X						
Pregnancy test [13]	X						
12-Lead ECG [14]	X			X	X (before surgery)	X	
Echocardiograph[15]	X	Tested before C3, C5, and C7			X (before surgery)	X	
Pathological examination of primary lesion	X				X (before surgery)		
RCB score					X (after surgery)		

	**Screening period**	**Neoadjuvant therapy**			**Surgery**	**End of Study/Withdrawal**	**Safety Follow-up** 25
	D−14 to D−1	C1–C8			Within 4 weeks (> 2 weeks) after C8		
		D7 ± 3	D21 ± 3	D21 ± 3			
Pathological response rate assessment					X (after surgery)		
Imaging Assessments [16]	X	Tested before C3, C5, and C7			X (before surgery)		
Chest CT	X				X (before surgery)		

Progression/Death		X			
Chidamide[17]		20 mg, po, bid, po, 2 weeks on, 1 week off, 8 cycles in total			
Epirubicin[18]		90 mg/ m^2, ivgtt, d1, q3w			
Cyclophosphamide[19]		600 mg/ m^2, ivgtt, d1, q3w			
Docetaxel[20]		100 mg/m^2, ivgtt, d1, q3w			
Drug dispensing/return[21]		X		X	

Comments:

Subjects who have failed previous screening are not allowed to be re-screened in this study (i.e., subjects who have signed the informed consent form again are not allowed to be re-screened; except for subjects who have failed screening due to age).

The following examinations should be completed within the time window (± 3 days) specified in the Schedule of Activities. In case of out-of-window examinations, the reason for out-of-window examinations should be recorded in the CRF. The investigator may increase the test items or increase the frequency of visits according to the clinical situation of the subject. Except for pathological examination, the laboratory and auxiliary examination results involved in this study can be accepted for examination in another hospital, and the investigator will determine whether to accept the examination results.

* Screening period: Physical examination, vital signs, and laboratory tests (including hematology, urinalysis, hepatic and renal functions, blood lipids, blood electrolytes, fasting blood glucose, blood HCG, etc.) should be performed within 14 days before the start of study treatment; tumor imaging examination (breast, chest and abdomen enhanced CT or enhanced MRI, and bone scan), 12-lead ECG, and echocardiography should be performed within 14 days before the start of study treatment. For laboratory test results, imaging examination results (bone scan results obtained 2 months before signing the informed consent form are acceptable), 12-lead ECG, and echocardiography obtained within 14 days before signing the informed consent form, if they are sufficient to support the investigator's judgment on whether the subject meets the inclusion criteria, such examinations do not need to be repeated during the screening period, otherwise they need to be repeated during the screening period.

1.Demographics: including initials, gender, ethnicity, marital status, date of birth, height, and weight.

2.Medical history: Including past history of breast cancer and treatment history (clinical/pathological diagnosis, time of diagnosis, clinical/pathological staging, HER2/ER/PgR/expression; whether surgery was performed, whether neoadjuvant therapy was performed, whether adjuvant therapy was performed, and whether radiotherapy was performed), history of tumors other than breast cancer, history of smoking and drinking (whether smoking and drinking, whether quitting), history of drug allergy (drug name, allergic symptoms), past diseases or concomitant diseases/symptoms (name, duration, whether treatment and outcome of diseases/symptoms), and stool habits (frequency).

3.Concomitant medications: Concomitant medications from the screening period to the end of study treatment should be recorded. Concomitant medication records should include the drug name, dose, route of administration, frequency of administration, purpose of administration, and start and end dates. If a subject starts a new systemic anti-tumor treatment during the safety follow-up period, only concomitant medications/treatments for treatment-related AEs will be recorded.

4.Physical examination: including evaluation of head, lymph nodes, eyes, ears, nose, throat, skin, oral cavity, musculoskeletal system, respiratory system, cardiovascular system, abdomen, genitourinary system, nervous system, and mental state. Performed once during the screening period, before surgery, and at the end of study/withdrawal (if not performed within the past 7 days).

5.Vital signs: Including respiratory rate, pulse, heart rate, blood pressure, and body temperature. Performed once during screening, on the last day of each cycle during neoadjuvant therapy, before surgery, and at the end of study/withdrawal (if not performed within the past 7 days).

6.Adverse events: Adverse events, concomitant medications/treatments, and unscheduled examinations should be documented in detail from the signing of the informed consent form until at least 21 days after the last dose, until the adverse events are resolved or reach a stable level that is considered irrelevant by the investigator, or the subject starts a new anti-tumor treatment or is lost to follow-up.

7.Hematology: including hemoglobin, red blood cell count, platelet count, white blood cell count, neutrophil count, and lymphocyte count. Performed once during screening, on days 7, 14, and 21 of each cycle of neoadjuvant therapy, before surgery, and at the end of study/upon withdrawal (if not performed within the past 7 days). If necessary, the investigator may increase the frequency of examinations according to the clinical conditions of the subject.

8.Blood biochemistry: including sodium, potassium, chlorine, calcium, phosphorus, magnesium, cholesterol, triglycerides, glucose, BUN or urea, creatinine, uric acid, total protein, albumin, alkaline phosphatase, ALT, AST, GGT, total bilirubin, and direct bilirubin. Blood biochemistry tests should be performed during the screening period, on D14 and D21 of Cycle 1 of neoadjuvant therapy, on the last day of each subsequent cycle, before surgery, and at the end/upon withdrawal (if not performed within the past 7 days). If necessary, the investigator may increase the frequency of examinations according to the clinical situation of the subject

9.Coagulation function test: including INR, APTT, PT, FIB, and TT. Performed once during the screening period and before surgery. During the study, the investigator will decide whether to perform the examination based on clinical practice or needs.

10.Urinalysis: including urine protein, urine glucose, urine red blood cells, and urine white blood cells. Performed once during the screening period, before surgery, and at the end/upon withdrawal (if not performed within the past 7 days). During the study, the investigator will decide whether to perform the examination based on clinical practice or needs. If the urinalysis shows that the urine protein is ++ or above, please add a 24-h urine protein test.

11.Routine stool test: including fecal occult blood. Once during the screening period, before surgery, and at the end of treatment/withdrawal (if not performed within the past 7 days). During the study, the investigator will decide whether to perform the examination based on clinical practice or needs.

12.Infectious disease screening: including 5-item hepatitis B test, HIV antibody, and HCV antibody test. Performed once during screening. If the results of hepatitis B and C tests are abnormal, the investigator should determine whether to perform viral replication (HBV-DNA, HCV-RNA) tests.

13.Pregnancy test: Female subjects of childbearing potential must undergo a blood HCG test to rule out pregnancy during the screening period, before surgery, and at the end of treatment/withdrawal (if not performed within the past 7 days).

14.12-lead ECG: Performed once during the screening period, on the last day of each cycle in the neoadjuvant therapy period, before surgery, and at the end of study/upon withdrawal (if not performed within the past 7 days). If the QTc interval increases by > 30 msec from baseline, or if the absolute value of QTc interval is ≥ 480 msec in any given ECG measurement, 2 additional ECG examinations (at least 10 minutes apart) are required.

15.Echocardiography: Performed once during the screening period, once at the end of every 2 cycles during the treatment period, and once before surgery. If symptoms such as chest pain and palpitations and ECG abnormalities are observed during the study, additional tests may be performed as appropriate.

16.Tumor imaging evaluation: The imaging evaluation will be performed according to the principles of RECIST v1.1. The screening period includes breast, chest, abdomen, and bone scans. Imaging examinations of the brain, neck, pelvis, and other sites can be added to rule out metastasis based on the clinical situation. MRI can be used for breast tumor assessment through CT or MRI. During the neoadjuvant therapy period, an assessment should be performed at the end of 2 cycles, 4 cycles, 6 cycles, and before surgical treatment under the same conditions as those of the baseline examination (use of contrast agent, etc.) (bone scan is performed once at the end of neoadjuvant therapy). Unscheduled imaging examinations can be performed when PD is suspected (such as worsening of symptoms).

17.Chidamide: 20 mg, po, bid, po, 2 weeks on, 1 week off, 8 cycles in total

18.Epirubicin: 90 mg/ m^2, intravenous injection, once every 21 days, calculated based on the actual body weight before each dose, and used in Cycles 1–4.

19.Cyclophosphamide: 600 mg/m^2, intravenous injection, d1, once every 21 days. The dosage should be calculated based on the actual body weight before each dose and used in cycles 1–4.

20.Docetaxel: 100 mg/m^2, intravenous injection, d1, once every 21 days; the dose should be calculated based on the actual body surface area before each dose, and used in cycles 5–8.

21.Drug dispensation/return: Except for drug dispensation in cycle 1 and drug return at the end of treatment, drugs were returned and dispensed at the end of each cycle, and corresponding records were made.

22.Safety follow-up: until at least 21 days after the last dose of the investigational drug, the investigator may choose clinical evaluation measures such as hematology, blood biochemistry, ECG, echocardiography, physical examination, vital signs, and KPS score according to the specific conditions of the subject as unscheduled examination records. Treatment-related AEs should be followed up until resolved, returned to baseline levels ≤ Grade 1, reaching a steady state, or reasonably explained (e.g., loss to follow-up, death).

List of Abbreviations

Abbreviations	Full name
AE	Adverse event
AJCC	American Joint Committee on Cancer
ALT	Alanine aminotransferase/alanine aminotransferase
ANC	Absolute neutrophil count
ASCO	American Society of Clinical Oncology
AST	Aspartate aminotransferase/aspartate aminotransferase
BUN	Blood Urea Nitrogen
CAP	College of American Pathologists
CxDy	Cycle x Day y
C_{max}	Maximum plasma concentration
CrCl	Creatinine clearance
CR	Complete Response
CRA	Site Manager
CT	Computed Tomography
CSCO	Chinese Society of Clinical Oncology
CTCAE	Common Terminology Criteria for Adverse Events
DCR	Disease Control Rate
ECG	Electrocardiogram
CRF	Electronic Case Report Form
EDC	Electronic Data Capture
GCP	Good Clinical Practice
h	hours
Hb	Hemoglobin
HBsAg	Hepatitis B Virus Surface Antigen
HBV	Hepatitis B virus
HC	Heavy chain
HCV	Hepatitis C virus

Abbreviations	Full name
HIV	Human Immunodeficiency Virus
ICF	Informed Consent Form
ICH	International Council for Harmonization of Technical Requirements for Pharmaceuticals for Human Use
IRB	Institutional Review Board
IV	Vein
JAMA	Journal of the American Medical Association
KPS	Cartridge Functional Status Scoring Criteria
LHRH	Gonadotropin-releasing hormone
MedDRA	Medical Dictionary for Regulatory Activities
MRI	Magnetic Resonance Imaging
NCI	National Cancer Institute
NMPA	National Medical Products Administration
ORR	Objective Response Rate
PD	Pharmacodynamics
PFS	Progression-free survival
PLT	Platelets
PR	Partial Response
po	Oral
QTcF	QT interval corrected for heart rate by Fridericia's formula
Q3W	Every Three Weeks
RBC	Erythrocytes
RCB	Residual tumor burden
RECIST	Response Evaluation Criteria in Solid Tumors
SADR	Serious Adverse Drug Reactions
SAE	Serious Adverse Events
SD	Stable disease
SOP	Standard Operating Procedures
SUSAR	Suspected Unexpected Serious Adverse Reaction

**Abbreviations**	**Full name**
$T_{1/2}$	Elimination half-life
TBIL	Total bilirubin
TEAEs	Treatment-Emergent Adverse Events
TMB	Tumor mutation burden
TSH	Thyroid Stimulating Hormone
ULN	Upper limit of normal
WBC	Leukocytes

1. BACKGROUND

Breast cancer is the most prevalent malignant tumor in women, accounting for 24.2% of all malignant tumors. In 2018, 2.09 million people were diagnosed with breast cancer worldwide, and about 630,000 people died of breast cancer. The prevalence of breast cancer in China accounts for 17.1% of all malignant tumors in women. In 2015, there were about 304,000 cases and about 70,000 deaths. Approximately 75 percent of patients with breast cancer are HR positive. Neoadjuvant therapy is an important treatment for stage II-III breast cancer. It can downstage the tumor and improve the chance of operable and breast-conserving surgery. Clinical studies have shown that patients with early breast cancer have similar prognosis after neoadjuvant therapy and postoperative adjuvant therapy. In addition, neoadjuvant therapy can also early determine the drug sensitivity and drug resistance in vivo, providing an excellent biological model for efficacy evaluation in the adjuvant treatment of breast cancer.

HR+/HER2- neoadjuvant therapy for breast cancer is mainly based on anthracycline combined with paclitaxel chemotherapy. Active neoadjuvant chemotherapy can only achieve pCR in about 10% of patients with HR-positive breast cancer. The emergence of new targeted drugs has brought new vitality and hope to patients with HR-positive breast cancer. The neoMONARCH and PALLET studies compared the efficacy of CDK4/6 inhibitors combined with aromatase inhibitors (AI)with single-agent endocrine therapy. The results showed that the combination with CDK4/6 inhibitors could significantly reduce the tumor cell proliferation index Ki67 and the apoptotic indexc-PARP, and achieve cell cycle arrest in more patients. However, the pCR did not increase, and the residual tumor burden (RCB) 0/1 did not significantly improve. The LORELEI study explored the efficacy of tislelizumab combined with letrozole as neoadjuvant therapy, and found thattislelizumab improved ORR in the overall population, most notably in the PIK3CA mutation population, but there was no significant difference in pCR rate between the overall population and the PIK3CA mutation population. Neoadjuvant endocrine therapy still requires continuous exploration, especially when targeted drugs are rapidly updated and iterated, and combined targeted therapy has become a new research direction to further improve the efficacy of neoadjuvant endocrine therapy.

Basic research shows that abnormal status of epigenetics is related to drug resistance, metastasis, and recurrence of tumors. Histone deacetylase (HDAC) is an important regulator of

epigenetic regulation, and targeting HDAC drugs provides a novel strategy for tumor treatment. Chidamide is an oral subtype-selective HDAC inhibitor independently developed in China. It is a novel epigenetic drug belonging to Class 1.1 new drugs and the first approved by the China Food and Drug Administration (CFDA) in China for the treatment of relapsed or refractory peripheral T-cell lymphoma (PTCL). The mechanism of action of Chidamide: mainly targets the 1,2,3 subtypes in the I class HDAC and the 10 subtype in the IIb class. It not only regulates abnormal epigenetic functions of tumors and induces and activates novel cellular immune functions, but also overcomes chemotherapy resistance and epithelial-mesenchymal transdifferentiation (EMT). In addition, chidamide's unique multiple mechanisms of action can improve the tumor microenvironment, thereby bringing long-term survival benefits to patients. The ACE study evaluated the clinical efficacy and safety of chidamide in combination with exemestane in patients with HR positive HER2 negative advanced breast cancer. This is also the first large-scale clinical study of epigenetic modulators in the treatment of solid tumors. The study enrolled postmenopausal patients with HR positive /HER2-negative advanced breast cancer resistant to endocrine therapy. All enrolled patients were randomly assigned in a 2:1 ratio to receive chidamide with exemestane (n=244) or placebo with exemestane (n=121). Results: The median PFS was 7.4 months in the Chidamide group and 3.8 months in the placebo group (investigator-assessed); the median PFS was 9.2months in the Chidamide group and 3.8 months in the placebo group (IRC-assessed). The ACE study suggested that for patients with HR- positive HER2- negative advanced breast cancer who have relapsed or progressed after endocrine therapy, the selective HDAC inhibitor chidamide combined with endocrine therapy can significantly improve their survival benefits, providing a new treatment option for these patients. Based on good accessibility and the ACE study, the 2020 and 2021 CSCO breast cancer clinical diagnosis and treatment guidelines listed chidamide combined with exemestane as Class 1A evidence of HR positive advanced breast cancer treatment after failure of tamoxifen/toremifene treatment and failure of non-steroidal AI treatment, Class I recommendation.

As an epigenetic modulator, chidamide has been shown in vitro and in vivo to induce apoptosis and growth arrest in cancer cells, reverse epithelial-mesenchymal transition and drug resistance in cancer cells, and enhance natural killer cells and antigen-specific CD8+ T lymphocyte-mediated anti-tumor activity. Chidamide was approved in China for the treatment of relapsed or refractory PTCL based on the results of a multicenter phase II trial. Real-world studies

(RWS) of chidamide in a large patient population further demonstrated good efficacy and potential survival benefits of chidamide in combination with chemotherapy in a relapsed or refractory setting, with the main toxicity associated with chidamide treatment being an acceptable hematologic adverse event.

In summary, we envision that neoadjuvant treatment with chidamide combined with chemotherapy will provide a better strategy for patients with HR+/HER2- breast cancer.

2 STUDY OBJECTIVES AND ENDPOINTS

2.1 Study Objective:

To explore the efficacy and safety of chidamide combined with chemotherapy as neoadjuvant therapy for stage II-III HR+/HER2- breast cancer.

2.2 Endpoints:

Primary endpoints:

RCB score of 0–1;

Secondary endpoints:

pCR (ypT0/is, ypN0);

bpCR (ypT0/is);

Objective response rate (ORR);

Breast conserving rate;

Safety endpoints:

For adverse events (AEs) and serious adverse events (SAEs), refer to NCI-CTCAE 5.0.

3 STUDY DESIGN

This study adopts a multi-center, single-arm, prospective design, and patients with stage II-III HR+/HER2- breast cancer were planned to be enrolled. The primary objective is to observe the efficacy and safety of chidamide combined with chemotherapy as neoadjuvant therapy in the treatment of stage II-III HR+/HER2- breast cancer. Breast MRI and other imaging examinations

will be repeated every 2 cycles to evaluate the efficacy. If the efficacy is confirmed, surgery will be performed within 4 weeks after cycle 8 of neoadjuvant therapy. If the efficacy is SD+/PD, the subject should withdraw from the study. If the toxicity is intolerable, the subject withdraws the informed consent or the investigator judges that the drug must be discontinued. The imaging evaluation will be performed according to RECIST 1.1 criteria, and the evaluation results obtained by the study site are the final results. Within 4weeks after surgical treatment, histopathological examination will be performed on the excised tumor tissues and lymph nodes (including the pathology of tumor margins); the pathological sections will be uniformly sent to the leading site for unified review.

Efficacy follow-up: All subjects must be followed up until tumor progression, death, or withdrawal of informed consent, whichever occurs first.

Safety follow-up: Subjects should be followed up until they start to receive other anti-tumor treatments; all AEs return to 0-1 degrees Celsius or baseline levels, or death, whichever occurs first.

Survival follow-up: All subjects will be followed up for survival until death, withdrawal of informed consent, or end of the trial, whichever occurs first.

4. Selection and withdrawal of subjects

4.1 Inclusion Criteria

Subjects must meet all of the following inclusion criteria to be enrolled in this study:

1. Female patients aged ≥ 18 and ≤ 75years old

2. All patients were histopathologically confirmed estrogen receptor (ER) positive (>10%) and HER2 receptor negative. Follow the 2018 ASCO-CAP HER2 Negative Interpretation Guideline criteria;

3. Previously untreated patients with stage II-III whose tumors meet the criteria ofAJCC version 8;

4. KPS score≥ 70;

5. The functional level of the organ must meet the following requirements:

(3) Bone Marrow Function

- ✓ ANC ≥ 1.5 × 10^9/L (no growth factors used within 14 days),
- ✓ PLT ≥ 100 × 10^9/L (without corrective treatment within7 days),
- ✓ Hb ≥ 100 g/L (without corrective treatment within7 days);

(4) Liver and kidney function

- ✓ TBIL ≤ 1.5 × ULN;
- ✓ ALT and AST ≤ 3 × ULN;
- ✓ BUN and Cr ≤ 1.5 × ULNand creatinine clearance ≥ 50 mL/min (Cockcroft-Gault formula);

6. Able to accept needle biopsy;

7. Voluntarily join the study, sign the informed consent, have good compliance and are willing to cooperate with follow-up.

4.2 Exclusion Criteria

Subjects with any of the following conditions are not eligible to participate in this clinical study:

1. Previously received other anti-tumor treatments of any form (chemotherapy, radiotherapy, molecular targeted therapy, endocrine therapy, etc.);

2. Concurrent use of any other anti-tumor treatment;

3. Bilateral breast cancer, inflammatory breast cancer, or occult breast cancer;

4. Stage IV breast cancer;

5. Breast cancer without histopathological diagnosis;

6. Patients with other malignancies within the past 5 years, except for cured cervical carcinoma in situ;

7. Patients with severe heart, liver, kidney and other important organ dysfunction;

8. Inability to swallow, chronic diarrhea, and intestinal obstruction, with multiple factors affecting drug intake and absorption;

9. Participated in other drug clinical trials within 4 weeks prior to enrollment;

10. Subjects with a known history of allergy to the drug components of this protocol; a history of immunodeficiency, including positive HIV test, HCV, active viral hepatitis B or other acquired or congenital immunodeficiency diseases, or a history of organ transplantation;

11. Any history of heart disease, including: (1) arrhythmia requiring medication or with clinical significance; (2) myocardial infarction; (3) cardiac failure; (4) any other heart disease judged by the investigator to be unsuitable for participating in this trial;

12. Pregnant or lactating women, women of childbearing potential with positive baseline pregnancy test results, or women of childbearing potential who are unwilling to take effective contraceptive measures during the entire study;

13. Presence of accompanying diseases that may pose serious risks to the safety of the patient or may affect the patient's ability to complete the study as judged by the investigator (including but not limited to severe hypertension, severe diabetes, and active infection that cannot be controlled by drugs);

14. History of clear neurological or mental disorders, including epilepsy or dementia. Any other condition that the investigator considers the patient unsuitable for participating in this study.

4.3 Withdrawal Criteria

4.3.1 Criteria for Subject Withdrawal

Withdrawal from this clinical study:

1. The subject voluntarily withdraws the informed consent form at any time;

2. Serious violation of inclusion/exclusion criteria after randomization;

3. Medical imaging progression or clinical progression;

4. Any clinical AEs, laboratory abnormalities, or other medical conditions indicating that the subject can no longer benefit from the treatment;

5. Occurrence of pregnancy during the study;

6. Those who seriously violate the trial protocol and the investigator evaluates that the treatment should be discontinued;

7. Other reasons as determined by the investigator.

4.3.2 Handling of withdrawn subjects

The investigator can suggest or provide new or alternative treatments to patients based on their actual conditions.

4.4 Discontinuation Criteria

The termination criteria for this study include but are not limited to the following:

1. Discovery of unexpected, significant, or unacceptable risks to subjects;

2. Major errors in the protocol found during the implementation of the study;

3. The study drug/trial treatment is ineffective, or it is meaningless to continue the trial;

4. Extreme difficulty in completing the trial due to reasons such as severe delay in subject enrollment or frequent protocol deviations.

If the principal investigator prematurely terminates or suspends this clinical study, he/she must inform the subject immediately, and report to the Ethics Committee in writing with the specific reasons.

4.5 Definition of End of Study

The end of the study is defined as when the last subject completes the surgery or the investigator believes that the study should be terminated early.

5 STUDY DRUGS

5.1 Study Drug Name and Strength

Chidamide tablets: off-white tablets, 5 mg/tablet. Developed and provided by Shenzhen Chipscreen Biosciences, Ltd. Sealed and stored below 25 °C, protected from light.

Epirubicin, cyclophosphamide, and docetaxel are all commercially available.

5.2 Drug Management

Drugs will be dispensed on the first day of each cycle during the treatment period. All investigational products must be stored in a safe and GCP-compliant place at each study site and managed by designated personnel (receipt, storage, dispensing, and recovery of drugs).

Investigators responsible for drug management need to record

The number of drugs dispensed and returned by each patient at each follow-up visit and the corresponding follow-up date. The entry and exit of all investigational products at each site should be recorded (including unused drugs and packaging). Patients must return all unused drugs and empty packages to the investigator at each follow-up visit, and return them to the sponsor for accountability and destruction after the end of the trial.

5.3 Method of Use

Patients with stage II-III HR+/HER2- breast cancer who meet the trial criteria will be enrolled from the date of the study.

Chidamide combined with EC-T chemotherapy, 21-day cycles, 4 cycles EC followed by 4 cycles T, 8 cycles in total

Chidamide: 20 mg, po, bid, po, 2 weeks on, 1 week off, 8 cycles in total
Epirubicin 90 mg/m^2, ivgtt, d1
Cyclophosphamide 600 mg/m^2, ivgtt, d1
Q3w, 4 cycles
Sequential
Docetaxel 100 mg/m^2, ivgtt, d1
Q3w, 4 cycles

Chidamide tablets: orally administered twice a week, 4 tablets/dose (20 mg/dose), with an interval of no less than 3 days (such as Monday and Thursday, Tuesday and Friday, Wednesday and Saturday, etc.), starting on the day before chemotherapy and 30 min after breakfast.

All patients continued treatment until the following occurred: disease progression, intolerable

toxicity, death, withdrawal from treatment, withdrawal of informed consent, or loss to follow-up.

The above medications can be adjusted according to the subject's adverse reactions in the protocol. Subjects continued medication until surgery, withdrawal of informed consent, or when the physician believed that medication must be discontinued. The dosing cycle is determined from the date of the subject's first dose. In case of any interruption of oral administration, missed dose, or vomiting during the study, the subject continues to take the drug in cycles according to the protocol, without making up the dose or adjusting the dose regularly. However, the details should be recorded in the source data: If a dose is missed, the time of the missed dose and the reason for the missed dose should be recorded in detail; if the dose is missed due to adverse drug reactions and other reasons, it should be recorded in the original medical record.

5.3 Treatment of Common Adverse Reactions and Dose Modification

The administration of the investigational product may be modified or delayed according to the adverse reactions of the subjects in the protocol. If dose adjustment is required for toxic effects occurring during the study, it is recommended to adjust the drug with greater correlation before adjusting the drug with lesser correlation. The reason for dose modification or delay, measures taken, and results should be recorded in the subject's medical record and case report form (CRF). The severity of AEs will be graded using the CTCAE v5.0 grading and evaluation system.

The efficacy of anti-tumor drugs is continuous. If the treatment is interrupted/delayed within the specified time limit, there is no evidence that the efficacy will be significantly reduced. Therefore, if the subject only interrupts/delays the treatment with some drugs related to toxicity, other drug treatments that are not interrupted/delayed still need to be carried out as planned. When the toxicity is basically recovered, all drug treatments should be carried out as planned to avoid disrupting the combined treatment regimen, and strive to give all drug treatments within the specified time window of each dosing cycle.

The dosing window is ± 3 days from the scheduled dosing date (calculated based on the first dose date). If the dosing window is exceeded (3 days), the dosing will be considered as a delayed dose, and the subsequent dosing dates will be recalculated based on the actual date of the last dose. If a delay is required due to toxicity (not clearly related to which drug), all drugs should be delayed at the same time if the criteria for re-administration can be met within 1 week. When resuming

administration, the entire combination regimen should be given in the order of administration specified in the original protocol as much as possible in principle. Chemotherapy is allowed to be suspended for up to 9 consecutive weeks, and chemotherapy will be discontinued if it exceeds 9 weeks. For situations not clearly specified in the protocol, the investigator should consider the benefit/risk ratio of the subject before making a decision. Under the circumstances clearly stipulated in the protocol, the drug must be interrupted and the dose must be reduced as required. Once the dose is reduced, it cannot be adjusted back unless some drugs are discontinued.

5.3.1 Management and dose modification of hematological adverse reactions

Dose modification of Chidamide

A routine blood test should be performed before the use of this product. Only when the relevant indicators meet the following conditions can the medication be started: absolute neutrophil count ≥ 1.5 × 10^9 /L, platelet count ≥ 75 × 10^9 /L, and hemoglobin ≥ 9.0 g/dL.Regular routine blood tests (usually once a week) are required during treatment.

Interrupt camrelizumab for Grade 3 or 4 neutropenia (neutrophil count < 1.0 × 10^9 /L). Grade 3 neutropenia with body temperature greater than 38.5°C or Grade 4 neutropenia should be treated with cytokines such as G-CSF. Routine blood test should be performed regularly (once every other day or at least twice a week) until the absolute neutrophil count returns to ≥ 1.5 × 10^9 /L, and after two consecutive examinations are confirmed, the treatment with this product can be continued: If the previous adverse reaction is Grade 3, the original dose or the dose can be reduced to 10 mg/time when the medication is resumed; if the previous adverse reaction is Grade 4, the dose should be reduced to 10 mg/time when the medication is resumed.

For grade 3 or 4 thrombocytopenia (platelet count < 50.0 × 10^9/L), interrupt camrelizumab and give interleukin-11 or thrombopoietin (TPO); if platelet count < 25.0 × 10^9 /L or bleeding tendency occurs, blood component transfusion should be considered.Routine blood test should be performed regularly (once every other day or at least twice a week) until platelet count returns to ≥ 75.0 × 10^9 /L, and after two consecutive examinations are confirmed, the treatment with this product can be continued: If the previous adverse reaction is Grade 3, the original dose or the dose can be reduced

to 10 mg/time when the medication is resumed; if the previous adverse reaction is Grade 4, the dose should be reduced to 10 mg/time when the medication is resumed.

Grade 3 or 4 anemia (hemoglobin decreased to < 8.0 g/dL): Interrupt camrelizumab and treat with erythropoietin (EPO); if hemoglobin is < 5.0 g/dL, provide blood transfusion. Routine blood test should be performed regularly (once every other day or at least twice a week). The treatment with this product can be continued after the hemoglobin returns to ≥ 9.0 g/dL and is confirmed by two consecutive tests: If the previous adverse reaction is Grade 3, the original dose or the dose can be reduced to 10 mg/time when the medication is resumed; if the previous adverse reaction is Grade 4, the dose should be reduced to 10 mg/time when the medication is resumed.

After treatment and dose reduction for the above hematological adverse reactions, if Grade 4 hematological adverse reactions occur again or Grade 3 neutrophil count decreased with body temperature higher than 38.5 °C, the treatment with this product should be discontinued.

Table 1. Dose modification of Chidamide Tablets in the case of Grade ≥ 3 hematological adverse events

**AE Category**	**AE grade**	**Dose Modification or Withdrawal#**
**Hematology Adverse event**	Grade 3*	Continue treatment and maintain the original dose
		Continue treatment with 2 tablets/dose reduction
		Interrupt medication, and resume medication afterGrade ≤ 1 AE within 2 weeks,<br>Maintain the original dose or reduce the dose 2 tablets/times
	Grade4 (or Grade 3 neutropenia with body temperature> 38.5°C)	Interrupt medication, resume medication after Grade ≤ 1 AE within2 weeks, reduce the dose by 2 tablets/

*, the treatment method was selected according to the medical judgment of the investigator.

#, Patients should withdraw from the trial if any of the following occurs:

Grade 4 hematologic adverse events or Grade 3 neutropenia with body temperature > 38.5°Cdespite dose reduction of Chidamide Tablets/Placebo due to hematologic adverse events;

Chidamide tablets were discontinued for more than 2 consecutive weeks.

Dose Modifications for Chemotherapy

According to the prescribing information and local treatment standards, dose modification and interruption of chemotherapy drugs are permitted due to toxic effects. The maximum interval of delay is 9 weeks (calculated from the actual time of the last dose), otherwise the chemotherapy should be discontinued (except for subjects who may benefit from continued treatment as judged by the investigator).

Dose modification references (or modifications performed by the investigator according to routine clinical practice) are provided below. If the dose of the drug is reduced due to toxic reactions, the dose should not be adjusted back during subsequent treatment. For those who cannot tolerate 2 dose reductions due to toxicity, the chemotherapy drug should be permanently discontinued.

Table 2. Dose modification levels of chemotherapeutic drugs

AE	Epirubicin	Cyclophosphamide	Docetaxel
Febrile neutropenia<br>Grade 4 neutropenia<br>Grade 4 thrombocytopenia	Two dose reductions are permitted, 25% each	Two dose reductions are permitted, 25% each	Two dose reductions are permitted, 25% each

The adverse reactions of epirubicin and cyclophosphamide are not completely consistent. They are adjusted according to the correlation between adverse reactions and the drug. If the adverse reactions are highly closely related, they should be adjusted first. If the adverse reactions are lowly related, one chemotherapeutic drug can be adjusted, or two chemotherapeutic drugs can be adjusted

in combination. The specific adjustments should be made according to the investigator's judgment and refer to common clinical practice.

5.3.2 Management and dose modification of non-hematological adverse reactions

Dose modification of Chidamide

If Grade 3 non-hematological adverse reactions occur, the drug should be interrupted and symptomatic treatment should be given. The physician should regularly examine and monitor relevant items according to the specific adverse reactions. Chidamide can be resumed when the adverse reactions are relieved to ≤ grade 1, but the dose should be reduced to 10 mg/time. If Grade ≥ 3 adverse reactions occur again after dose reduction, Chidamide treatment should be discontinued.

Treatment should be discontinued if Grade 4 non-hematologic adverse reactions occur during administration.

See Table 1 for the short form of dose modification of Chidamide Tablets.

Table 3. Dose modification of Chidamide Tablets in the presence of Grade ≥ 3 AEs.

AE Category	AE grade	Dose Modification or Withdrawal#
**Non-hematologic Adverse Events**	Grade 3 *	Interrupt medication, and resume medication afterGrade ≤ 1 AE within 2 weeks,<br>Maintain the original dose or reduce the dose 2 tablets/times
	Grade4	Withdrawal from trial

*, the treatment method was selected according to the medical judgment of the investigator.

#, Patients should withdraw from the trial if any of the following occurs:

After the dose of chidamide tablets/placebo is reduced to 2 tablets/times (if it is the chidamide group, equivalent to a dose of10 mg), there are still≥ 3 grade adverse events potentially related to the investigational product;

Chidamide tablets were discontinued for more than 2 consecutive weeks.

Dose Modifications for Chemotherapy

Epirubicin combined with cyclophosphamide sequenced by docetaxel: Common therapeutic drug-related adverse reactions include: alopecia, neutropenia, sensory neurotoxicity, electrocardiogram abnormal, heart failure, severe myocardial injury, fatigue/asthenia, muscle pain/arthralgia, AST increased, alkaline phosphatase increased, anemia, nausea, infection, and diarrhea. Dose modifications are provided below (or modified by the investigator according to clinical practice). If the dose of the drug is reduced due to toxic reactions, the dose should not be adjusted back during subsequent treatment. For those who cannot tolerate 2 dose reductions due to toxicity, the chemotherapy drug should be permanently discontinued.

Table 4. Dose modification levels of chemotherapeutic drugs

AE category	AE grading	Epirubicin	Cyclophosphamide	Docetaxel
Non-hematologic Adverse Events	Grade 3*	Reduced by 25%	Reduced by 25%	Reduced by 25%
	Grade 4	Withdrawal from trial	Withdrawal from trial	Withdrawal from trial

*The treatment method was selected based on the medical judgment of the investigator.

5.3.3 Management of common adverse events

The comprehensive results of clinical trials of Chidamide monotherapy showed that Chidamide has good and controllable safety and tolerability indicators. The main adverse reactions observed in the completed clinical trials of Chidamide monotherapy are as follows:

Mainly hematological adverse reactions: including platelet count decreased, white blood cell or neutrophil count decreased, and hemoglobin decreased;

Other adverse effects include:

Systemic adverse reactions: including asthenia and fever;

Gastrointestinal adverse reactions: including diarrhea, nausea, and vomiting;

Metabolic and nutritional adverse reactions: including decreased appetite, hypokalemia, and hypocalcemia;

Others: including dizziness, rash, etc.

In international clinical trials of HDAC inhibitors, a similar drug to Chidamide, adverse reactions such as QTc interval prolongation, pericardial effusion, and deep vein thrombosis have been reported, but the correlation between these safety issues and Chidamide is currently unclear.

When an AE occurs during the study, the investigator should actively perform symptomatic treatment according to the actual clinical situation and routine clinical procedures, and record the concomitant treatment and medication in detail in the course of the disease and CRF. According to the data of preliminary clinical trials of Chidamide, Chidamide monotherapy has good tolerability. The investigator should carry out medical treatment according to the actual clinical situation. The following treatment methods are for reference:

Diarrhea: Before starting oral administration of the investigational product, the investigator should inform the subject in detail of the possibility of diarrhea and treatment measures for diarrhea. If diarrhea occurs, symptomatic treatment should be given first, with close follow-up or observation (≤ 14 days). It is clinically recommended to start oral administration of montmorillonite powder (3 g/packet, 3 times/day) on the day of diarrhea; for severe diarrhea, oral or intravenous infusion of electrolytes and loperamide can be given, and 1 capsule (2 mg) can be taken if each stool is unformed, with the highest dose of 8 capsules (16 mg)/day, until the diarrhea stops for more than 12 h. For Grade III diarrhea that still cannot be relieved or Grade I–II diarrhea with complications, it is recommended to interrupt chidamide first. The administration will be resumed after the AE returns to Grade 1.

Vomiting: Symptomatic treatment should be given first, with close follow-up. If the time of vomiting is close to the time of administration on that day, the time of vomiting should be recorded in detail. However, regardless of whether vomiting affects the absorption of the investigational drug, the drug should be continued to be taken according to the cycle specified in the protocol without make-up doses or cycle adjustments.

Rash: Symptomatic treatment should be given first, and close follow-up should be conducted.

Recommended symptomatic and supportive treatment: strengthen skin care, keep the skin clean, avoid secondary infections; avoid pressure or friction; use moisturizers or lubricants, topical use of lotions or lubricants containing urea and corticosteroids; topical use of antifungal or antibiotic treatment if necessary.

Hypokalemia and hypocalcemia: The investigator will provide symptomatic fluid replacement or observation according to the conditions of the subject and the AE, and increase the frequency of blood biochemistry tests according to clinical needs.

Cardiotoxicity: QT prolongation: Subjects should be corrected for hypokalemia, hypomagnesemia, or hypocalcemia before starting chidamide. Patients should be vigilant for the use of Chidamide in the following situations: 1) underlying cardiac diseases or special circumstances: such as previous cumulative high-dose anthracycline treatment; 2) congenital long QT interval syndrome; 3) hypokalemia, hypocalcemia, and hypomagnesemia; 4) concomitant use of 2 or more drugs that prolong the QT interval.

Decreased left ventricular ejection fraction (LVEF): Before starting chidamide treatment, it should be confirmed that LVEF is within the normal range. LVEF should be monitored regularly during the study to ensure that LVEF is not below the lower limit of normal.

When a Grade 3 non-hematological adverse reaction occurs under combined medication, the doctor should judge the correlation between the adverse reaction and the related drug according to the specific adverse reaction; if the adverse reaction is related to the drug combined with chidamide, the corresponding treatment and dose adjustment should be made with reference to the package insert of the drug.

6. Concomitant medications

During the trial, in addition to receiving the investigational drug, patients can receive corresponding supportive treatment for adverse events that occur. No other anti-tumor treatment should be given during the study to avoid affecting the efficacy evaluation of the investigational product. The specific provisions are as follows:

1) Throughout the study, patients are not allowed to use other anti-tumor measures other than the investigational product, including radiotherapy, chemotherapy, surgery, targeted therapy, and anti-

tumor traditional Chinese medicine.

2) The investigator can provide corresponding supportive treatment after evaluating the relationship between an AE and the investigational drug. Record the start and duration of supportive care in the CRF. These treatments include antiemetic, antidiarrheal, antipyretic, antiallergic, rash, use of antihypertensive drugs, use of analgesics, use of antibiotics, and others (eg, use of blood products).

3) Non-hematological toxicities can be treated with corresponding drugs at grade 2 and recorded in the concomitant medications.

4) Hematological toxicity can be treated with corresponding drugs at grade 3 and recorded in concomitant medications.

When the patient's hemoglobin is 5.0 g/dL to 8.0 g/dL, the investigator should decide whether to give erythropoietin (EPO) treatment; when the hemoglobin is < 5.0 g/dL, blood transfusions should be given.

When the platelet count of the patient is $< 50.0 \times 10^{9}$/L, interleukin-II or thrombopoietin (TPO) treatment is given; if the platelet count is $< 25.0 \times 10^{9}$/L or there is a tendency to bleed, blood transfusion is given.

When the neutrophil count of a patient is $< 1.0–0.5 \times 10^{9}$ /L without fever, the investigator should decide whether to give cytokine therapy such as G-CSF; if Grade 3 neutropenia with body temperature higher than 38.5 °C or Grade 4 neutropenia occurs, cytokine therapy should be given.

5) For patients who have been using bone modulators (such as bisphosphonates, RANK ligand drugs, etc.) before the screening period, the original drug and the original dose should be maintained during the trial; for patients who have not used bone modulators before the screening period, it is not recommended to add them during the trial.

6) The information on all concomitant medications (generic name, purpose of administration, dose, time of administration, etc.) received by the subjects should be completely recorded in the source data.

7. Study Procedures

Before the start of the study, patients must read and sign the current informed consent form

approved by the ethics committee (EC). All study procedures must be carried out within the time window specified in the study schedule.

7.1 Screening Period

After signing the informed consent form, the subjects will enter the screening period. Unless otherwise stated, the following screening procedures must be completed within 14 days prior to randomization:

Demographics: initials, gender, ethnicity, marital status, date of birth, height, and weight;

General medical history: Past medical history and treatment history (clinical/pathological diagnosis, time of diagnosis, clinical/pathological staging,HER2/ER/PR/expression, surgery or not, neoadjuvant therapy or not, progression to recurrent/metastatic breast cancer), smoking and drinking history (frequencyrate, amount, and duration), drug allergy history (drug name, allergic symptoms), past diseases or concomitant diseases/symptoms (disease/ symptom name, concomitant medication name, dose, method of administration, and outcome); stool habits (frequency);

Physical Examination: Includes evaluation of the head, eyes, ears, nose, throat, skin, skeletal muscles, respiratory organs, cardiovascular, gastrointestinal, genitourinary, and nervous systems. Abnormalities at baseline will be recorded in the CRF;

Vital signs: include respiratory rate, pulse, systolic and diastolic blood pressure (subject is in a sitting position), and body temperature. Vital sign results should be obtained and assessed prior to each dose of study treatment.

KPS score;

Hematology: Including hemoglobin, red blood cell count, platelet count, white blood cell count, and neutrophil count.

Blood biochemistry: including sodium, potassium, chlorine, cholesterol, triglycerides, glucose, BUN or urea, creatinine, total protein, albumin, alkaline phosphatase, ALT, AST, GGT, total bilirubin, and direct bilirubin.

Coagulation function test;

Urinalysis: Including urine protein, urine glucose, and urine occult blood; performed 1 time during

the screening period and before surgery; if the urinalysis shows that the urine protein is++or above, please add 24-h urine protein quantitation;

Routine stool test: Including fecal occult blood;

Infectious disease screening: hepatitis B panel, HIV antibody, and HCV antibody tests;

Pregnancy test: Female subjects of childbearing potential must undergo a blood HCG test to rule out pregnancy during the screening period;

12-lead ECG: 3 ECG examinations (at least 10 min apart) are required, and the mean of the 3 QTc intervals will be used as the baselineQTc interval;

Echocardiography: Reported within 14 days prior to randomization (including qualified echocardiography completed prior to the signing of ICF);

Tumor hormone receptor status,

Peripheral blood sample collection: 8 mL of anticoagulant blood (intention);

Tumor histopathological sampling: Tumor tissue samples were collected through puncture biopsy during the screening period. All study sites were responsible for collecting samples, and the Ki67, apoptosis marker cPARP, and MT were measured by Shengjing Hospital of China Medical University;

Tumor imaging examination: Including breast, chest, abdomen, and bone scans. Imaging examinations of the brain, neck, pelvis, and other sites may be added to rule out metastasis based on clinical findings. MRI can be used for breast tumor assessment through CT or MRI. The investigator may add scan sites at baseline or during subsequent tumor assessments as clinically indicated. Imaging results such as CT/MRI scans obtained before signing the informed consent form can be used for tumor assessment during the screening period as long as they meet the requirements (and within 21 days before the first dose of the investigational product);

Concomitant medications/treatments: Concomitant medications from the screening period to the end of study treatment should be recorded;

Follow-up of adverse events: Recorded from the day when the subjects sign the informed consent form until at least 14 days after the last dose; after completing all the above screening assessments,

eligible subjects will start to receive the investigational product.

7.2 Treatment Period

Tumor assessments were performed every 2 cycles during the treatment period. If a patient withdraws from the study due to reasons other than PD (such as AEs and management reasons), tumor assessments should be continued once every 8 weeks until PD or the start of a new anti-tumor treatment (whichever occurs first). The investigator may increase the test items or increase the frequency of visits according to the clinical situation of the subject.

Vital signs: Checked on the last day of each cycle during the neoadjuvant therapy period after enrollment and before surgery;

Hematology: On D7, D14, and D21 of Cycle 1 of neoadjuvant therapy after enrollment, and once before surgery;

Blood biochemistry: On D14 and D21 of Cycle 1 of neoadjuvant therapy after enrollment, once before surgery;

Coagulation function: Once before surgery;

Peripheral blood sample collection: 8 mL of anticoagulant blood (intention) will be collected at baseline and at the end of every 2 cycles of treatment;

Tumor histopathological sampling: Samples will be collected via needle biopsy at cycle 2 (intention-to-treat), at the first efficacy evaluation (after 2 cycles of treatment after grouping), at the time of PD/SD (intention-to-treat), at the second efficacy evaluation (after 4 cycles of treatment after grouping), at the time of PD/SD (intention-to-treat), and at surgery. All study sites were responsible for collecting samples, and the Ki67, apoptosis marker cPARP, and MT were determined by Shengjing Hospital of China Medical University;

12-lead ECG: Performed once at the end of each cycle; during the study, if the QTc interval increases by > 30 msec from baseline, or if the absolute value of QTc interval is ≥ specified in any specified ECG measurement, 2 additional ECG examinations are required (at least 10 min apart).

Echocardiography: Additional examinations may be performed as appropriate if symptoms such as chest pain and palpitations or ECG abnormalities are observed during the study;

Tumor imaging examination: The tumor imaging examination time point during the treatment period was determined after the start of the study treatment (i.e., C1D1), regardless of the time when the administration was interrupted due to toxic reactions during this period. The time window allowed for tumor imaging examination is ± 7 days. Assessments will be performed at the end of Cycle 2 and Cycle 4. If imaging-confirmed PD occurs, the subject should discontinue the study treatment. No other anti-tumor treatment can be performed before PD;

Concomitant medications/treatments: Concomitant medications/treatments during the study should be recorded at any time;

Adverse events: Adverse events during the study should be observed and recorded at any time;

Progressive disease/death: Progressive disease during the study and the time and specific situation of death should be observed and recorded at any time.

7.3 Patient Perioperative

The subject continued medication until the subject underwent surgery. The following examinations should be performed and recorded before surgery: physical examination, vital signs, KPS score, blood routine, blood biochemistry, peripheral blood sample collection, coagulation function test, urinalysis, stool routine, pregnancy test, ECG, and tumor imaging assessment. During the procedure, tumor samples are collected by excision. All study sites are responsible for collecting samples, measuring the levels of Ki67 and MT by Shengjing Hospital of China Medical University, checking bpCR, pCR, and RCB scores after surgery, and recording adverse events and concomitant medications in real time.

7.4 End of Study Treatment/Withdrawal from Study

The subjects continued the treatment until the completion of surgery, 4 cycles of treatment after changing the protocol due to disease progression, intolerable toxicity, withdrawal of informed consent, or the investigator judged that the treatment must be discontinued.

At the end of the study treatment or upon withdrawal from the study, if the subject has not undergone the examinations (except for echocardiography and tumor imaging assessment) within 7 days before the end of the study, the following examinations should be performed:

KPS score;

Vital signs;

Physical examination;

Routine blood test: if not performed within 7 days before;

Urinalysis: If not performed within 7 days prior;

Routine stool test: If not performed within the past 7 days;

Blood biochemistry: if not performed within the past 7 days;

Pregnancy test;

12-lead ECG: If not performed within the past 7 days;

Tumor imaging examination: If not performed within the past 8 weeks;

Concomitant medication/treatment: recorded in real time;

Adverse events: recorded in real time;

Drug recovery: The remaining drugs were recovered.

7.5 Efficacy Evaluation

7.5.1 Imaging assessment

Contrast-enhanced MRI is recommended for tumor imaging evaluation. Subjects with a history of contrast media allergy will be managed according to the guidelines for the prevention of contrast media allergy at the study site to perform enhanced MRI whenever possible. Color Doppler ultrasound evaluation is permitted if the subject is strictly contraindicated to contrast agents.

Imaging assessments will be performed according to the principles of RECIST v1.1. The screening period includes breast, chest, abdomen, and bone scans. Imaging examinations of the brain, neck, pelvis, and other sites can be added to rule out metastasis based on the clinical situation. The investigator may add scan sites at baseline or during subsequent tumor assessments as clinically indicated. Imaging results such as CT/MRI scans obtained before signing the informed consent form can be used for tumor assessment during the screening period as long as they meet the requirements (and within 21 days before the first dose of the investigational product).

Subsequent imaging assessments should be performed under the same conditions as those of the baseline examination (slice thickness, use of contrast agent, etc.). The tumor imaging examination time points during the administration period were determined after the start of the study treatment, and the administration suspension did not change the evaluation time. The time window allowed for tumor imaging examination is ± 7 days. The specific assessment time points are as follows:

The first assessment is performed on Day 21 of Cycle 2, the second assessment is performed on Day 21 of Cycle 4, the third assessment is performed on Day 21 of Cycle 6, and the fourth assessment is performed on Day 21 of Cycle 8.

According to RECIST 1.1, bone scan and PET are not suitable for target lesion efficacy evaluation. When necessary, these tests may be performed less frequently for non-target lesions if they are used to assess such lesions. For example, bone scan may be repeated only when CR is confirmed in target lesions or when progression of bone lesions is suspected.

7.5.2 Primary endpoints

RCB score of 0–1

7.5.3 Secondary endpoints

Efficacy endpoints:

1.pCR (ypT0/is, ypN0)

2. bpCR (ypT0/is)

3.ORR

4. Breast conserving rate;

8 SAFETY EVALUATION

The safety of the investigational product was evaluated during the study period through AE records (including SAEs), laboratory tests, vital signs, physical examination, KPS score, echocardiography, and ECG records. Subjects should be closely observed for symptoms and signs after administration during the trial. Adverse events/reactions should be effectively handled in a

timely manner to ensure the safety and interests of the subjects. The type, symptoms, time of occurrence, degree (or grade), symptomatic treatment, and outcome of adverse drug events/reactions should be recorded after timely and effective treatment, and then the adverse events should be analyzed, evaluated, and statistically analyzed as the basis for the continuation of the trial.

8.1 Physical Examination and Vital Signs

The study physician is responsible for physical examination, including general conditions, skin and mucous membranes, lymph nodes, head and neck, chest, abdomen, musculoskeletal system, nerve reflexes, respiratory system, cardiovascular system, genitourinary system, and mental state.

Vital signs include the following: respiratory rate, pulse, heart rate, blood pressure, and body temperature.

The KPS score is evaluated by the study doctor according to the KPS scoring criteria.

8.2 Laboratory Tests

The following laboratory parameters will be tested at the time points specified in the "Schedule of Activities". Unscheduled clinical laboratory tests may be performed at any time for subject safety reasons.

Table5. Laboratory test requirements.

Blood routine	Blood biochemistry	Urinalysis
Hemoglobin<br>Erythrocytes<br>Leukocytes<br>Neutrophil count<br>Lymphocyte count<br>Platelet count	Total bilirubin<br>Direct bilirubin<br>ALT<br>AST<br>Alkaline phosphatase<br>r-GT<br>Total protein<br>Albumin<br>Urea Nitrogen<br>Uric acid<br>Creatinine<br>Blood Glucose<br>Potassium<br>Sodium<br>Chlorine<br>Calcium<br>Phosphorus<br>Magnesium	Urine protein<br>[a] Urine glucose<br>Urine red blood cells<br>Urine leukocytes
**Coagulation function**	**Infectious disease screening**	**Other**

INR<br>APTT<br>PT<br>FIB<br>TT	Hepatitis B panel<br>HIV antibody<br>HCV antibody	Pregnancy Test [b]

Note: a. If the semi-quantitative method shows that the urine protein is ++ or above, a quantitative 24-h urine protein test will be performed.

b. Women of childbearing potential must undergo a blood HCG test to rule out pregnancy during the screening period, and a urine HCG test can be performed at other time points.

8.3 Electrocardiogram

12-lead ECG will be performed by a qualified physician at the time points specified in the Schedule of Activities. All ECG examinations should be performed after the subjects have rested in a quiet position for at least 10 min. Contents include at least: heart rate, QT, QTc, and P-R interval. Three tests (at least 10 min apart) are required during the screening period, and the mean value of the three QTcF results will be used as the baseline QTcF. To assess subject safety, the study doctor will compare the ECG results with the baseline tests. If the QTc interval increases by > 30 msec from baseline, or if the absolute value of QTc interval is ≥ specified in any specified ECG measurement, 2 additional ECG examinations should be performed at least 10 min apart to determine the accuracy of the original measurement and to rule out abnormal ECGs caused by incorrect wire placement. If the QTc value read by the machine is prolonged, according to the above description, if the qualified physician determines that the QTc value is within the acceptable range, repeated measurements may not be performed.

8.4 Echocardiography

Echocardiography will be performed by a qualified physician at the time points specified in the Schedule of Activities. During the administration of the investigational product, the study doctor will assess and monitor LVEF at the time points specified in the protocol. During administration,

subjects with symptoms of heart failure or clinically significant decreased LVEF should be treated and monitored according to standard medical guidelines as judged by the investigator, and a cardiologist should be consulted when necessary. In the event of clinically uncontrollable symptoms of severe heart failure (NYHA Class III or IV) or significant decrease in LVEF (below the lower limit of normal or below 50%), the investigational product should be discontinued according to the requirements in Section 5.4 - Dose Modification Protocol, and treatment and monitoring should be continued according to standard medical guidelines.

8.5 Adverse Events (AE)

8.5.1 Definition of Adverse Events

An adverse event (AE) refers to any untoward medical occurrence in a clinical trial subject after signing the informed consent form, which does not necessarily have a causal relationship with the investigational product. An AE can be any unexpected and unfavorable symptom, sign, disease, or abnormal test result, regardless of whether it is related to the investigational drug. AEs include the following: 1) medical conditions/diseases existing before the start of study treatment, which are only recorded as AEs if they worsen after the start of study treatment; 2) any new AEs; 3) abnormal changes in laboratory test results, which are only considered clinically significant.

8.5.2 Criteria for the severity of adverse events

Refer to NCI-CTCAE 5.0 for the grading criteria of adverse drug events.

Refer to the following criteria for AEs not listed in NCI-CTCAE 5.0:

Grade I: mild; asymptomatic or mild clinical symptoms; clinical or diagnostic observations only; intervention not indicated.

Grade II: Moderate; minimal, local, or non-invasive intervention indicated; limiting age-appropriate instrumental activities of daily living (IADL). Instrumental activities of daily living refer to preparing meals, shopping, using the telephone, managing money, etc.

Grade III: Severe or medically significant but not immediately life-threatening; hospitalization or prolongation of hospitalization indicated; disabling; limiting self-care ADL. Self-care activities of daily living refer to bathing, dressing and undressing, feeding self, using the toilet, taking medications, and not bedridden.

Grade IV: Life-threatening consequences; urgent intervention indicated.

Grade V: Death related to AEs.

8.5.3 Causality assessment criteria

AEs include all unexpected clinical manifestations. As long as these events occur after the signing of the informed consent form, they should be reported as AEs regardless of whether they are related to the investigational product, whether the subject is assigned to the investigational product group, or whether the subject has received the drug. Any discomfort complained by the subject or abnormal changes in objective laboratory measurements during the treatment should be truthfully recorded. The severity, duration, measures taken, and outcome of the AE should also be noted. The investigator should comprehensively determine the relationship between the AE and the investigational product, and assess the possible relationship between the AE and the investigational product according to the following five categories: "definitely related, possibly related, unlikely related, not related, and indeterminable".

"Definitely related", "possibly related", and "indeterminable" events were all listed as adverse drug reactions. When calculating the incidence of adverse events, the total of these two categories was used as the numerator and the total number of subjects used for safety evaluation was used as the denominator. The criteria are shown in Table 6:

Table 6. Causality assessment criteria.

Grading	Acceptance criteria
Definitely related	The occurrence of the event follows a reasonable temporal sequence after drug administration, the event follows a known response pattern to the suspected drug, improves after drug discontinuation, and the event recurs after repeated administration.
possibly related	The occurrence of the event follows a reasonable temporal sequence from administration of the drug, the event does not follow a known response pattern to the suspected drug, and the event may also be caused by the patient's clinical status or other treatments.

Unlikely related	The occurrence of the event does not follow a reasonable temporal sequence from administration of the drug, the event does not follow a known response pattern to the suspected drug, and the event may also be caused by the patient's clinical status or other treatments.
Not related	The occurrence of the event does not follow a reasonable temporal sequence from administration of the drug, the event does not follow a known response pattern to the suspected drug, the event may also be caused by the patient's clinical status or other treatment methods, the event resolves after the disease improves or other treatment methods are discontinued, and the event occurs after repeated use of other treatment methods.
Unassessable	There is no clear relationship between the occurrence of the event and the time sequence after drug administration. The event is similar to the known reaction type of the drug. Other concomitant medications may also cause the corresponding event.

8.5.4 Recording and reporting of AEs

All AEs should be documented in detail by the investigator, including: description of the AE and all related symptoms, time of onset, severity, duration, measures taken, and final outcome (recovered/resolved, with or without sequelae, resolved, unchanged, death, unknown). In this study, drug safety should be assessed from the day of signing the informed consent form until 28 days after the last dose. All adverse events (serious and non-serious) should be recorded on the adverse event report page of the case report form and should be reported using accurate medical terminology.

Related AEs: Follow-up is required until any of the following occurs:

1) Disappear or improve to baseline level;

2) The event was assessed again as not related to Chidamide;

3) Death;

4) Initiation of new anti-tumor treatment regimen;

5) The investigator confirms that no further improvement is expected and the patient's condition is

stable;

6) No further clinical data or final database will be collected.

Unrelated AEs: Follow-up is required until any of the following occurs:

1) Disappear or improve to baseline level;

2) The severity improves to within grade 1;

3) Death;

4) Initiation of new anti-tumor treatment regimen;

5) The investigator confirms that no further improvement is expected;

6) No further clinical data or final database will be collected.

8.6 Serious Adverse Event (SAE)

8.6.1 Definition of serious adverse event

Serious adverse event (SAE) refers to a medical occurrence during the clinical trial that results in hospitalization, prolonged hospitalization, disability, incapacity, life-threatening or death, or congenital malformation. An adverse event that meets one or more of the following criteria is an SAE:

✓ Leading to death;

✓ Life-threatening (defined as when the subject is at immediate risk of death at the time of the event);

✓ Requiring hospitalization or prolonged hospitalization;

✓ Resulting in permanent or severe disability/incapacity;

✓ Resulting in congenital anomalies or birth defects;

✓ Important medical events: These AEs may not be life-threatening, result in death, or require hospitalization. However, based on medical judgment, they may jeopardize the subject and require medical or surgical intervention to prevent any of the above outcomes.

The investigator should report all SAEs in a timely manner, including clinical diagnosis, management, and outcome, and follow up until they return to normal, resolve, or stabilize. At the same time, detailed records should be made in the original medical records, and the SAE report form should be filled out.

8.6.2 Potential drug-induced liver injury

Drug-induced liver injury will be considered if abnormal ALT and/or AST levels are accompanied by abnormal elevation of total bilirubin level, and the following conditions are met without other causes of liver injury. Such cases should always be considered as important medical events.

Table 7. Evaluation criteria for potential drug-induced liver injury

<table>
<tr><td>Baseline</td><td colspan="2">Normal (ALT/AST and total bilirubin)</td><td colspan="2">abnormal (ALT/AST and total bilirubin)</td></tr>
<tr><td rowspan="2">Treatment</td><td>ALT⩾3×ULN</td><td>AST⩾3×ULN</td><td>ALT or AST⩾2× baseline with a value ⩾3×ULN</td><td>ALT or AST⩾8× ULN</td></tr>
<tr><td colspan="2">Meet either of the above requirements with total bilirubin ⩾2×ULN and alkaline phosphatase ⩽2×ULN without hemolysis</td><td colspan="2">Meet either of the above requirements with total bilirubin ⩾1×ULN or value >3× ULN</td></tr>
</table>

Subjects should return to the study site for evaluation as soon as possible (preferably within 48 hours) after being informed of the abnormal results. The evaluation should include laboratory tests, detailed medical history, and physical assessment, and should consider the possibility of liver tumors (primary or secondary).

In addition to repeating ALT and AST tests, laboratory tests should also include albumin, creatine kinase, total bilirubin, direct and indirect bilirubin, γ-glutamyltransferase, prothrombin time (PT)/international normalized ratio (INR), and alkaline phosphatase. Further testing may include testing for acute hepatitis A, B, C, and E and liver imaging.

Detailed medical history should include: history of alcohol use, acetaminophen, soft drugs, various supplements, family medical history, occupational exposure, sexual behavior, travel, contact with patients with jaundice, surgery, blood transfusion, liver disease or allergic disease, etc.

If repeated tests confirm that the above laboratory criteria are met, the possibility of potential drug-induced liver injury should be considered in the absence of other causes of abnormal liver function tests, and it is not necessary to wait for all liver function etiological test results. Such cases of potential drug-induced liver injury should be reported as SAEs.

8.6.3 Progressive disease

Progressive disease (including progressive signs and symptoms) should not be reported as an SAE. However, death due to progressive disease during the study or safety reporting period, i.e., within 28 days (inclusive) after the last dose of the investigational product, should be reported as an SAE. Hospitalization due to symptoms and signs of disease progression should not be reported as an SAE. During the study or safety reporting period, if the final outcome of the cancer is death, the event leading to death must be reported as an SAE.

8.6.4 Hospitalization

AEs resulting in hospitalization or prolonged hospitalization during the clinical study should be considered as SAEs. Any initial admission to a health care facility (even if less than 24 hours) meets this criterion.

The following hospitalizations do not constitute an SAE:

✓ Rehabilitation facility

✓ Nursing home

✓ Routine emergency admission

✓ Day surgery (e.g., outpatient/day/ambulatory surgery)

✓ Hospitalization or prolonged hospitalization unrelated to an AE is not an SAE. For example:

✓ Hospitalization due to pre-existing diseases without new AEs or worsening of pre-

existing diseases (e.g., to examine laboratory abnormalities that have persisted before the study);

- ✓ Hospitalization for management reasons (e.g., annual physical examination);
- ✓ Hospitalization during the clinical trial as specified in the trial protocol (e.g., according to the requirements of the trial protocol);
- ✓ Elective hospitalization unrelated to AEs (e.g., elective cosmetic surgery);
- ✓ Scheduled treatment or surgery should be documented throughout the study protocol and/or in the subject's individual baseline information;
- ✓ Hospitalization merely for blood product use.

Diagnostic or therapeutic invasive (e.g., surgery) and non-invasive procedures should not be reported as AEs. However, when the disease condition leading to this operation meets the definition of an AE, it should be reported. For example, acute appendicitis occurring during the AE reporting period should be reported as an AE, and the resulting appendectomy should be recorded as the treatment for the AE.

8.6.5 Reporting system of serious adverse events

Serious adverse events should be reported from the signing of the informed consent form until the end of the safety follow-up period. In the event of an SAE, whether it is an initial report or a follow-up report, the investigator must complete the "Serious Adverse Event (SAE) Report Form" immediately, with a signature and date, and notify the sponsor and the Drug Safety Department of Shenzhen Chipscreen Biosciences Co., Ltd. within 24 h of knowing of the event. Relevant authorities must be informed of the SAE in a timely manner according to regulatory requirements (see Table 5. SAE reporting methods).

SAEs that occur after the safety follow-up period and are suspected to be related to the investigational product should be collected. SAEs should be documented in detail, including symptoms, severity, causality with the investigational product, time of onset, time of treatment, measures taken, time and method of follow-up, and outcome. If the investigator believes that an SAE is not related to the investigational product but potentially related to the study conditions (such as the termination of the original treatment, or comorbidities during the trial), the relationship should

be explained in the description section of the SAE report form. If the severity of an ongoing SAE or its relationship with the investigational drug changes, a follow-up report should be submitted immediately. If the investigator believes that a previously reported SAE is misreported, it can be corrected, revoked, or downgraded in the follow-up report and reported according to the SAE reporting procedure.

The email address for SAE reporting to the Drug Safety Department of Shenzhen Chipscreen Biosciences Co., Ltd. is:pv@chipscreen.com.

Table 8. SAE reporting method

Units	Contact	Fax/Telephone/Address
Shengjing Hospital of China Medical University	Ethics Committee	Tel./Fax:024-9661510027<br>Email:llwyh@sj-hospital.org
Shenzhen Chipscreen Biosciences, Ltd.	Drug Safety Department	pv@chipscreen.com
Department of Drug Registration, National Medical Products Administration		1. Fax: 010-8363228 (preferred)<br>2. Email:yjjdc@nmpa.gov.cn (In case of fax failure, the mailbox and PDF format should be used, and a request receipt should be set and filed. yjjdc = Office of Study Oversight)<br>3. EMS courier: Department of Drug Registration, National Medical Products Administration, North Xiayuan, Zhanlan Road, Xicheng District, Beijing, China (if the mail is still unsuccessful, please inform EMS)<br>Tel.: 010-88331134
Medical Safety and Blood Division, Bureau of Medical Administration, National Health Commission of the People's Republic of China		Fax: 010G8792734 (preferred)<br>Email:saefex@163.com ("Available mailboxes" must be in PDF format when fax fails)<br>Tel.: 010-68792201
Drug regulatory authorities of provinces, autonomous regions, and municipalities	Refer to the reporting requirements of the drug regulatory authorities of all provinces, autonomous regions, and municipalities directly under the Central Government	

8.6.6 Pregnancy

If a female subject becomes pregnant during the clinical trial, the subject should withdraw from the study.

The investigator should follow up the outcome of the pregnancy until 1 month after delivery.

If a subject experiences an SAE during pregnancy, the "NMPA Serious Adverse Event Report Form" should be completed and reported according to the time limit and requirements for SAEs.

If the outcome of the pregnancy is stillbirth, spontaneous abortion, or fetal malformation, it is considered an SAE, and the "NMPA Serious Adverse Event Report Form" should also be completed and reported according to the time limit and requirements for SAEs.

9 STUDY MANAGEMENT

9.1 Ethics and Informed Consent

9.1.1 Ethics

This clinical trial must comply with the Declaration of Helsinki (2008), NMPA's Good Clinical Practice (GCP), and relevant regulations. Before the start of the trial, approval must be obtained from the ethics committee of the study site. During the clinical study, certain amendments to the study protocol should be reported to the Ethics Committee and filed. The principal investigator is responsible for submitting the interim report of the trial regularly according to the relevant requirements of the ethics committee. After the end of the trial, the principal investigator should inform the ethics committee that the trial has been completed.

9.1.2 Informed consent

Subjects must give informed consent to participate in this study before receiving the investigational drug to protect their legal rights and interests. The investigator is responsible for providing the subjects or their designated representatives with a complete and comprehensive introduction to the objectives of this study, the effects of the drug, possible toxic and side effects, and possible risks. The subjects should be informed of their rights, risks, and benefits. Conversation is a very important part of the informed consent process. If the subject and his/her legal representative are illiterate, the informed consent process should be attended by a witness. After the subject or his/her legal representative verbally agrees, the witness should sign the informed consent form. The witness's signature should be on the same day as the subject's signature. The informed consent form should indicate the version number and version date.

9.2 Protocol Amendments

Any necessary changes to the protocol should be made in the form of protocol amendments, and should be submitted to the Ethics Committee for approval or filing after being signed and approved by the principal investigator. The details of previous amendments should also be described in the protocol.

9.3 Data Management

9.3.1 Data Collection

In this study, the CRF is used for study data collection; the study site personnel should receive systematic training.

9.3.2 Data management and quality control

In order to ensure the authenticity and reliability of the clinical trial data and improve the quality of clinical data, the CRA will review the integrity, consistency, and accuracy of the trial data in the clinical database according to the standard operating procedures during the trial, and guide the personnel of the study institution to make necessary supplements or corrections to the problematic data. If the clinical personnel or data management personnel have any questions about the data, they should raise queries to the study personnel or data entry personnel. Relevant personnelmust respond to the queries and make corrections or explanations to the problematic data. If necessary, multiple queries can be raised until the problematic data is resolved. The medical personnel and data management personnel should perform consistency comparison of SAEs on a regular basis.

At the end of the study, the data manager and medical personnel willperform final quality control on all data, summarize all protocol deviations and violations during the study, and hold a data verification meeting. Only after all data meet the quality requirements can the study statistician perform data analysis.

9.3.3 Data review and monitoring of the study institution

Before the initiation of the study, the study director should introduce the study protocol and CRF. During the trial, the person in charge of data monitoring should regularly check the completeness of patient records, the accuracy of CRF entries, the compliance with the trial protocol and GCP, and the progress of enrollment to ensure that the investigational product is stored,

dispensed, and counted according to the regulations. During this period, key study personnel must be able to assist data monitoring personnel.

The investigator must keep the source documents of each subject participating in the study, including study medical records and visit records (inpatient or outpatient medical records), including demographic parameters and medical information, laboratory data, ECG, and the results of any other examinations or evaluations. All information on the CRF must be derived from the source documents in the patient's file. The investigator must also keep the informed consent form signed by the patient. The investigator must confirm that all relevant source documents can be monitored to verify that they are consistent with the CRF. The monitoring criteria require 100% monitoring of the obtained informed consent forms, compliance with inclusion/exclusion criteria, SAE records, and data required for the evaluation of all primary endpoints and safety endpoints. Additional checks were performed on the consistency of the source data and CRFs according to the monitoring plan specified in the trial. Any information about the patient's identity in the source documents will not be disclosed.

9.4 Protocol Violations

All requirements specified in the study protocol must be strictly implemented. Any intentional or unintentional deviation from or violation of the study protocol and GCP principles can be classified as a protocol deviation or violation. If a protocol deviation is found, the investigator should fill in the protocol violation record, record the time of discovery, time and process of the event, cause, and corresponding treatment measures in detail, which should be signed by the principal investigator and reported to the Ethics Committee.

10 DATA ANALYSIS AND STATISTICAL METHODS

10.1 Sample Size Calculation

The primary objective of this study is to explore the efficacy and safety of chidamide combined with chemotherapy as neoadjuvant therapy in patients with stage II-III HR+/HER2- breast cancer. The primary endpoint is RCB 0-1. According to the literature and the historical data of the site, RCB 0-1 = 0.20 for conventional neoadjuvant chemotherapy. This combination regimen can be increased to 0.35, with a significance level of $\alpha = 0.05$ and a power of 80%. A Simon Minimax two-stage design is adopted: 31 subjects are required in the first stage, and the study will be terminated if ≤ 6

subjects reach RCB 0-1; otherwise, the study can proceed to the second stage, with 22 subjects required in the second stage. Considering a dropout rate of 10%, the total sample size of the two stages was 59.

10.2 Statistical Analysis Plan

10.2.1 Statistical analysis datasets

The analysis population in this study included the full analysis set (FAS), per-protocol set (PPS), and safety set (SS).

Full analysis set (FAS): The analysis set determined according to the intention-to-treat principle. All enrolled subjects with at least one administration record will be included in this analysis set. The FAS is the primary analysis set for the efficacy analysis of this study.

Per Protocol Set (PPS): a subset of the Full Analysis Set. All enrolled subjects with at least one administration record and without any major protocol violation that is considered to affect the efficacy analysis will be included in this analysis set.

The efficacy analysis of the investigational product was performed based on the FAS and PPS.

Safety set (SS): All enrolled subjects with at least one administration record will be included in the SS. The safety analysis of the investigational product was performed on the safety analysis set.

10.2.2 Statistical analysis methods

10.2.2.1 Basic method

In this study, unless otherwise stated, the data will be summarized using descriptive statistics according to the following general principles.

The measurement data will be summarized using mean, standard deviation, median, maximum, and minimum; the enumeration data will be summarized using frequency and percentage, and the 95% confidence interval of the percentage will be given if necessary; for time-event data, the survival rate will be estimated using the Kaplan-Meier method and the survival curve will be plotted.

10.2.2.2 Analysis of efficacy endpoints

The primary endpoint is the ORR rate, which will be analyzed based on FAS and PPS, with FAS as the primary analysis set.

Descriptive analysis of RCB score; pathological complete response (pCR); bpCR; breast conserving rate;

10.2.2.3 Safety analysis

All adverse events (AEs) will be coded using MedDRA and graded according to NCI CTCAE 5.0. A treatment-emergent adverse event (TEAE) is defined as any adverse event that newly occurs or worsens from baseline (before study treatment) after the start of study treatment.

The safety analysis will be mainly summarized using descriptive statistics. AEs, SAEs, Grade ≥ 3 AEs, Grade ≥ 3 SAEs, treatment-related AEs, treatment-related SAEs, AEs with an incidence of ≥ 5%, SAEs with an incidence of ≥ 5%, AEs leading to dose modification, and AEs leading to treatment discontinuation will be statistically summarized.

Normal laboratory test results before the study but abnormal after treatment and the relationship between the abnormal changes and the investigational product were described.

The differences in vital signs from baseline will be summarized by visit; the baseline and the highest post-baseline score of KPS will be summarized; the baseline and the worst post-baseline clinical abnormality grading of ECG and echocardiography will be summarized.

Safety analysis includes but is not limited to the above analyses.

10.2.3 Statistical software

SAS 9.4 or above will be used for analysis.

10.3 Dropouts

All subjects who have completed the informed consent form and passed the screening are entitled to withdraw from the clinical trial at any time. Subjects who have not completed at least 1 dose of the investigational product and are unable to undergo safety and efficacy evaluations are considered dropouts regardless of the time and reason for withdrawal (enrollment followed by disease progression with clear medical evidence is not considered a dropout). When a subject drops out, the investigator must record the reason for dropout, complete all possible assessments, and

carefully fill in the last visit record. Subjects who drop out due to adverse reactions and are finally judged to be related to the investigational product after follow-up should notify the investigator. Subjects who only underwent screening and withdrew from the study without obtaining a drug number were not considered as dropouts. If a subject has completed a complete cycle with detailed records, statistical analysis should be performed during safety evaluation.

Subjects who withdraw from the study cannot be re-enrolled in the study and their numbers cannot be used again.

Appendix I: Clinical Staging Criteria for Breast Cancer (AJCC 8th Edition, TNM Staging of Breast Cancer)

Stage 0	TisN0M0
Phase I	T1N0M0
Stage IIA	T0N1M0
	T1N1M0
	T2N0M0
Stage IIB	T2N1M0
	T3N0M0
Stage IIIA	T0N2M0
	T1N2M0
	T2N2M0
	T3N1, 2M0
Stage IIIB	T4N0M0, T4N1M0, T4N2M0
Stage IIIC	Any T, N3M0
Stage IV	Any T Any N, M1

Appendix II: Karnofsky Performance Status Score (KPS)

Status	Physical functioning level	Score
Able to move and work normally, no need for care	Normal, no signs or symptoms	100 points
	Able to carry on normal activities, with mild symptoms and signs	90 points
	Barely able to carry on normal activity with some signs or symptoms	80 points
Unable to work, limiting daily activities, needing care	Capable of all self-care but unable to carry out normal life and work	70 points
	Capable of most self-care but occasionally requires assistance	60 points
	Often in need of care	50 points
Unable to carry on any self-care; medical care required; progressive disease	Cannot carry on any self-care; requires special care and assistance	40 points
	Severely disabled	30 points
	Severe illness requiring hospitalization and aggressive supportive care	20 points
	Critical, imminent death	10 points
	Death	0 point

Appendix III: 2018 ASCO/CAP Guidelines for HER2 Detection in Breast Cancer

Subject	**2018 ASCO/CAP Breast Cancer HER2 Testing Guideline Recommendations**	
HER2 IHC Scoring Criteria	HER2 0 negative	No staining or ≤ 10% of infiltrating cancer cells show incomplete, weak cell membrane staining
	HER2 1+ negative	Incomplete, weak cell membrane staining in >10% of infiltrating cancer cells
	HER2 2+ Indeterminate	>10% of infiltrating cancer cells exhibit intact, weak to moderate membrane staining
	HER2 3+ positive	>10% of infiltrating cancer cells exhibit strong, complete, and uniform cell membrane staining
HER2 ISH Scoring Criteria	HER2/CEP17 ≧ 2.0 and mean HER2 gene copy/cell ≧ 6.0	ISH positive
	HER2/CEP17 ≧ 2.0 and mean HER2 gene copies/cell < 4.0	Combined IHC results:<br>If IHC 0/1+, consider HER2 negative and comment<br>If IHC 2+, another person who is not aware of the previous results should count at least 20 cells in the 2+ region. If the results are changed, after discussion and internal procedures, it should be finally determined that if the results remain unchanged, it will be considered as HER2 negative and annotated<br>HER2-positive if IHC 3+
	HER2/CEP17 < 2.0 and average HER2 gene copy/cell ≧ 6.0	Combined IHC results:<br>If IHC 0/1+, consider HER2 negative and comment<br>If IHC 2+, at least 20 cell counts in 2+ area by another person who is unaware of previous results, if results are changed, after discussion and other internal procedures<br>Final judgment was made later<br>If the result is unchanged, it is considered HER2 positive; if IHC 3+, it is considered HER2

		positive
	HER2/CEP17 < 2.0, normal<br>HER2 gene copy/cell ≧ 4.0 and <6.0	Combined IHC results:<br>If IHC 0/1+, consider HER2 negative and comment<br>If IHC 2+, another person who is not aware of the previous results should count at least 20 cells in the 2+ region. If the results are changed, a final judgment should be made after discussion and internal procedures. If the results remain unchanged, the subject will be considered HER2 negative and annotated<br>HER2-positive if IHC 3+
	HER2/CEP17 < 2.0, normal<br>HER2 gene copies/cell < 4.0	ISH negative

Appendix IV: Response Evaluation Criteria in Solid Tumors

Response Evaluation Criteria in Solid Tumors Version 1.1 (Excerpt)

(New Response Evaluation Criteria in Solid Tumors: Revised RECIST Version 1.1)

Note: This appendix is translated internally and is for reference only. Please refer to the English version during practice.

1. BACKGROUND

2. Purpose

3. Measurability of tumor at baseline

3.1 Definitions

At baseline, tumor lesions/lymph nodes will be categorized as measurable or non-measurable according to the following definitions:

3.1.1 Measurable disease

Tumor lesions: Must be accurately measured in at least one dimension (longest diameter in the plane of measurement is to be recorded) with a minimum size of:

- 10 mm by CT scan (CT scan slice thickness no greater than 5 mm)
- 10 mm caliper measurement by clinical exam (lesions which cannot be accurately measured with calipers should be recorded as non-measurable)
- 20 mm by chest X-ray
- Malignant lymph nodes: To be considered pathologically enlarged and measurable, a lymph node must be ≥ 15 mm in short axis when assessed by CT scan (CT scan slice thickness is not recommended)

More than

5 mm). At baseline and during follow-up, only the short axis will be measured and followed.

3.1.2 Non-measurable lesions

All other lesions, including small lesions (longest diameter < 10 mm or pathological lymph nodes with ≥ 10 mm to < 15 mm short axis) and truly non-measurable

Lesions. Lesions considered truly non-measurable include: meningeal disease, ascites, pleural or pericardial effusion, inflammatory breast cancer, lymphangitic involvement of skin or lung,

Abdominal masses and cystic lesions that cannot be confirmed or followed by imaging.

3.1.3 SPECIAL CONSIDERATIONS FOR LESION MEASUREMENT

Bone lesions, cystic lesions, and lesions previously treated with local
therapy require particular comment: Bone Lesions:

- Bone scan, PET scan, or plain films are not considered adequate imaging techniques to measure bone lesions, but may

be used to confirm the presence or disappearance of bone lesions.

- Lytic bone lesions or mixed lytic-blastic lesions, with identifiable soft tissue components, that can be evaluated by tomography techniques such as CT or MRI can be considered as measurable lesions if the soft tissue component meets the definition of measurability described above;

- Blastic bone lesions are non-measurable. Cystic Lesions:

- Lesions that meet the criteria for radiographically defined simple cysts should not be considered as malignant lesions because they are, by definition, simple cysts, neither measurable nor non-measurable;

- Cystic metastases can be considered as measurable lesions if they meet the definition of measurability described above. However, if non-cystic lesions are present in the same patient, these are preferred for selection as target lesions.

Lesions with prior local treatment:

- Lesions situated in a previously irradiated area, or in an area subjected to other loco-regional therapy, are usually considered non-measurable unless there has been demonstrated progression in the lesion. The study protocol should describe in detail the conditions under which these lesions are considered measurable.

3.2 Description of the measurement method

3.2.1 Lesion measurement

For clinical evaluation, all tumor measurements should be recorded in metric notation. All baseline evaluations should be performed as close as possible to the start of treatment must be completed within 28 days (4 weeks) before the start of treatment.

3.2.2 Evaluation method

The same technique and method should be used for baseline assessment and subsequent measurement of lesions. All lesions must be evaluated by imaging examinations except those that cannot be imaged but can only be evaluated by clinical examination.

Clinical lesions: Clinical lesions will only be considered measurable when they are superficial and ≥ 10 mm diameter as assessed using calipers (e.g., skin nodules). Pair

For subjects with skin lesions, it is recommended to use color photography including a ruler to measure the size of the lesion for archiving. When lesions are evaluated by both imaging and clinical examination, imaging evaluation should be performed since it is more objective and may be repeated at the end of the study.

Chest X-ray: Chest CT is preferred over chest X-ray when tumor progression is an important endpoint because CT is more sensitive than X-ray, especially for new lesions. Chest X-ray is only applicable when the boundary of the measured lesion is clear and the lungs are well ventilated.

CT and MRI: CT is currently the best available and reproducible method for efficacy evaluation. This guideline defines measurability of lesions on CT scan based on the assumption that CT slice thickness is ≤ 5 mm. If CT slice thickness is greater than 5 mm, the minimum size for a measurable lesion should be twice the slice thickness. MRI is also acceptable in certain situations (eg, for body scans).

Ultrasound: Ultrasound should not be used as a method of measuring lesion size. Ultrasound examination is not reproducible after the measurement is completed due to its operational dependence, and the identity of techniques and measurements between different measurements cannot be guaranteed. If new lesions are identified by ultrasound during the study, CT or MRI should be used for confirmation. If radiation exposure from CT is considered, MRI may be used instead.

Endoscopy, laparoscopy: The use of these techniques for objective tumor evaluation is not recommended, but they can be used when biopsy specimens are obtained

CR may also be used to confirm recurrence in trials where recurrence following CR or surgical resection is an endpoint.

Tumor markers: Tumor markers alone cannot be used to evaluate objective tumor response. However, if the marker level exceeds the upper limit of normal at baseline, it must return to normal when used to evaluate complete response. Because tumor markers vary by disease, this should be considered when including measurement criteria in the protocol. Specific criteria for CA-125 response (in recurrent ovarian cancer) and PSA response (in recurrent prostate cancer) have been published. In addition, the Gynecologic Cancer Intergroup has developed CA-125 progression criteria, which will be added to the objective tumor evaluation criteria for first-line treatment regimens for ovarian cancer.

Cytology/histology: These techniques can be used to differentiate between PR and CR in specific cases specified in the protocol (e.g., residual benign tumor tissue is often present in lesions of germ cell tumors). When effusions are a potential adverse effect of a therapy (e.g., treatment with taxane compounds or angiogenesis inhibitors) and the measurable tumor has met criteria for response or stable disease, the cytological confirmation of the neoplastic origin of any effusion that appears or worsens during treatment can be used to distinguish between response (or stable disease) and progressive disease.

4. Tumor Response Assessments

4.1 Assessment of Overall Tumor and Measurable Disease

In order to evaluate the objective response or possible future progression, it is necessary to perform a baseline assessment of the total tumor burden of all tumor lesions to provide a reference for the subsequent measurements. In clinical protocols where objective response is the primary endpoint, only subjects with measurable disease at baseline should be included. Measurable disease is defined as the presence of at least one measurable lesion. For trials where the primary endpoint is PD (time to progression or degree of progression at a fixed date), the protocol must specify whether enrollment is restricted to subjects with measurable lesions or whether subjects without measurable lesions are also eligible.

4.2 Baseline Documentation of Target and Non-Target Lesions

When more than one measurable lesion is present at baseline, all lesions up to a maximum of five lesions total (and a maximum of two lesions per organ) representative of all involved organs should be identified as target lesions and will be recorded and measured at baseline (this means in instances where subjects have only one or two organ sites involved, a maximum of two and four lesions respectively will be recorded)

Target lesions must be selected on the basis of size (longest diameter), be representative of all involved organs, and measurements must be reproducible. Sometimes

When the largest lesion cannot be measured reproducibly, the next largest lesion that can be measured reproducibly should be selected.

Lymph nodes require special attention because they are normal tissues and can still be detected by imaging even if there is no tumor metastasis. Pathological lymph nodes which are defined as measurable and may be identified as target lesions must meet the criterion of a short axis of ≥ 15 mm by CT scan. Only the short diameter should be tested at baseline. The short axis of the nodule is often used by radiologists to determine whether the nodule has metastasized. Nodal size is normally reported as two dimensions in the plane in which the image is obtained (for CT scan this is almost always the axial plane; for MRI the plane of acquisition may be axial, sagittal, or coronal). The minimum value is the short diameter. For example, an abdominal node which is reported as being 20 mm × 30 mm has a short axis of 20 mm and qualifies as a malignant, measurable node. In this example, 20 mm is the measurement of the nodule. Nodules with a diameter of ≥ 10 mm but < 15 mm should not be considered target lesions. Nodules < 10 mm are not considered pathological nodules and do not need to be recorded or further observed.

The sum of the calculated diameters of all target lesions (including the longest diameter of non-nodal lesions and the short diameter of nodal lesions) will be reported as the baseline sum diameters. If lymph node diameter is included,

as mentioned above, only the short diameter is included. The baseline sum of diameters will be used as a reference for the baseline level of the disease.

All other lesions, including pathological lymph nodes, can be regarded as non-target lesions and do not need to be measured, but should be recorded at baseline. If recorded as

"Present", "absent", or in rare cases "unequivocal progression". It is possible to record multiple non-target lesions involving the same organ as a single item on the case record form (e.g., multiple enlarged pelvic lymph nodes or multiple liver metastases).

4.3 Response Criteria

4.3.1 Target Lesion Assessment

Complete response (CR): Disappearance of all target lesions. Any pathological lymph nodes (whether target or non-target) must have reduction in short axis to < 10 mm.

Partial response (PR): At least a 30% decrease in the sum of diameters of target lesions, compared with baseline.

Progressive disease (PD): At least a 20% increase in the sum of diameters of target lesions, taking as reference the smallest sum on study (this includes the baseline sum if that is the smallest on study). In addition to the relative increase of 20%, the sum must also demonstrate an absolute increase of at least 5 mm.

(The appearance of one or more new lesions is also considered progression).

Stable disease (SD): Neither sufficient shrinkage to qualify for PR nor sufficient increase to qualify for PD, taking as reference the smallest sum diameters while on study.

4.3.2 Considerations for Target Lesion Assessment

Lymph nodes: Lymph nodes identified as target lesions should always have the actual short axis measurement recorded (measured in the same anatomical plane as the baseline examination), even if the nodes regress to below 10 mm. This means that when lymph nodes are included as target lesions, the sum of lesions may not be zero even if complete response criteria are met, since a normal lymph node is defined as having a short axis of < 10 mm. CRFs or other data collection methods may therefore be designed to have target nodal lesions recorded in a separate section where, in order to qualify for CR, each node must achieve a short axis < 10 mm. For PR, SD and PD, the actual short axis measurement of the nodes is to be included in the sum of target lesions.

Target lesions that become too small to measure: While on study, all lesions (nodal and non-nodal) recorded at baseline should have their actual measurements recorded at each subsequent evaluation, even when very small (e.g., 2 mm). However, sometimes CT scans may become so faint because they are too small that it is difficult for the radiologist to define the exact value and may be reported as "too small to measure". When this occurs, it is important that a value be recorded on the CRF. If it is the opinion of the radiologist that the lesion has likely disappeared, 0 mm should also be recorded. If a lesion is believed to be present and is faintly seen but too small to measure, a default value of 5 mm should be assigned. (Note: It is unlikely that this situation will occur in lymph nodes, because they generally have a measurable size under normal circumstances, or are often surrounded by adipose tissue as in the retroperitoneum; but if this situation occurs and cannot be measured, the default value is 5 mm). The default value of 5 mm is derived from the cutting thickness of the CT scan (this value is not due to the different cutting thickness of the CT degree values). Since there is little chance of repeated occurrence of the same measurement value, providing this default value will reduce the risk of incorrect evaluation. To reiterate, however, if the radiologist is able to provide an accurate measure of the lesion size, the actual value must be recorded, even if the lesion diameter is less than 5 mm.

Lesions that split or coalesce: When non-nodal lesions fragment, the longest diameters of the fragmented portions are added together to calculate the sum of the diameters of the lesions. Similarly, for bound lesions, the plane between the bound components distinguishes them and the maximal diameter is calculated. However, if the lesions have truly coalesced such that they are no longer separable, the longest diameter of the coalesced lesion as a whole should be taken as the longest diameter.

4.3.3 Evaluation of Non-Target Lesions

This section defines the tumor response criteria for non-target lesions. While some non-target lesions may actually be measurable, they do not need to be measured and can be assessed only qualitatively at the time points specified in the protocol.

Complete response (CR): Disappearance of all non-target lesions and normalization of tumor marker level. All lymph nodes were non-pathological in size (short axis

<10 mm).

Non-CR/Non-PD: Persistence of one or more non-target lesion(s) and/or persistence of tumor marker level above the normal limits. Progressive disease: Unequivocal progression of existing non-target lesions. Note: the appearance of one or more new lesions is also considered progression.

4.3.4 Special notes on the assessment of progression of non-target lesions

The definition of progression of non-target disease requires additional explanation as follows: When the subject has measurable non-target disease, to achieve unequivocal progression on the basis of the non-target disease, there must be an overall level of substantial worsening in non-target disease such that, even in presence of stable or partial response, the overall tumor burden has increased sufficiently to merit discontinuation of therapy. A modest increase in the size of one or more non-target lesions is usually not sufficient to qualify for progression. The designation of overall progression solely on the basis of change in non-target disease in the face of SD or PR of target disease will therefore be extremely rare.

When the subject's non-target lesions are all non-measurable: This circumstance arises in some phase III trials when it is not a criterion of study inclusion to have measurable disease. The same criteria apply here as noted above, since there are no measurable lesions in this setting. Because worsening in non-target disease cannot be easily quantified (by definition: if all lesions are truly non-measurable) a useful test that can be applied when assessing patients for unequivocal progression is to consider when the increase in overall disease burden based on the change in non-measurable disease is comparable in magnitude to the increase that would be required to declare PD for measurable disease. For example, an increase in tumor burden representing an additional 73% increase in volume (which is equivalent to a 20% increase diameter in a measurable lesion). Examples include an increase in a peritoneal effusion from "trace" to "large"; an increase in lymphangitic disease from "localized" to "widespread"; or may be described in protocols as "sufficient to require a change in therapy". Examples include an increase in a pleural effusion from trace to large, an increase in lymphatic involvement from the primary site to distant sites, or may be described in protocols as a "necessary change in treatment". If unequivocal progression is observed, the subject should be considered to have progressive disease overall at that time point. It is preferable to have objective criteria that can be applied to the assessment of non-measurable lesions. Note that the added criteria must be reliable.

4.3.5 New Lesions

The appearance of new malignant lesions denotes disease progression; therefore, some evaluation of new lesions is important. There are no specific criteria for the detection of lesions on imaging; however, the finding of a new lesion should be unequivocal. For example, progression cannot be attributed to differences in imaging techniques, changes in imaging modality, or findings thought to represent something other than tumor (for example, some new bone lesions may be simply healing or recurrence of pre-existing lesions). This is particularly important when the patient's baseline lesions show partial or complete response. For example, necrosis of a liver lesion may be reported on a CT scan report as a new cystic lesion, which it is not.

A lesion identified on follow-up but not on baseline examination will be considered a new lesion and will indicate disease progression. For example, if a subject is found to have visceral lesions at baseline and metastases are found during a CT or MRI brain scan, the subject's intracranial metastases will be considered as the basis for PD, even if he/she has not undergone a brain scan at baseline. If a new lesion is equivocal, for example because of its small size, further treatment and follow-up evaluation will be required to determine whether it represents a new lesion. If repeat scans confirm there is definitely a new lesion, then progression should be declared using the date of the initial scan. While FDG-PET response assessments of lesions generally require additional study, it is sometimes reasonable to incorporate the use of FDG-PET scanning to complement CT scanning in assessment of progression (particularly possible new disease). New lesions on the basis of FDG-PET imaging can be identified according to the following procedures:

Negative FDG-PET at baseline, followed by positive FDG-PET at follow-up, indicates disease progression. No FDG-PET at baseline and a positive FDG-PET at follow-up:

If the positive FDG-PET at follow-up corresponds to a new site of disease confirmed by CT, this is PD.

If the positive FDG-PET at follow-up is not confirmed as a new site of disease on CT, additional CT scans are needed to confirm the site of disease progression (if so, the date of PD will be the date of the initial abnormal FDG-PET scan).

If the positive FDG-PET at follow-up corresponds to a pre-existing site of disease on CT that is not progressing on the basis of the radiographic images, then the disease is not progressing.

4.4 Best overall response evaluation

The best overall response evaluation is the best response recorded from the start to the end of the trial, taking into account any necessary conditions for confirmation. Sometimes the response occurs after the end of treatment, so the protocol should clarify whether the efficacy evaluation after the end of treatment should be considered within the best overall efficacy evaluation. Protocols must specify how any new therapy before progression affects the best response. The best response of subjects mainly depends on the results of target and non-target lesions and the manifestation of new lesions. In addition, it also depends on the nature of the trial, protocol requirements, and outcome measures. Specifically, in non-randomized trials where response is the primary endpoint, confirmation of PR or CR is required to determine which is the best overall response.

4.4.1 Time Point Response

It is assumed that efficacy responses will occur at specific time points in each protocol. Table 1 provides a summary of the overall response at each time point for the subject population with measurable disease at baseline.

If the subject has no measurable lesions (no target lesions), refer to Table 2.

4.4.2 Missing Assessments and Not Evaluable Descriptions

If a lesion cannot be imaged or measured at a particular time point, the subject is not evaluable at that time point. If only a subset of lesion measurements are made at an assessment, usually the case is also considered NE at that time point, unless there is evidence that the contribution of the individual missing lesion(s) would not change the assigned time point response. This is likely to occur in the setting of disease progression. For example, if a subject had a baseline sum of 50 mm with 3 measured lesions and only 2 lesions were subsequently assessed, but those gave a sum of 80 mm, the subject will have achieved PD status, regardless of the contribution of the missing lesion.

4.4.3 Best overall response: all timepoints

The best overall response can be determined once all the data of the subject are available.

Best response determination in trials where confirmation of complete or partial response is not required: Best response in these trials is defined as the best response across all time points (for example, a subject who has SD at first assessment, PR at second assessment, and PD at last assessment has a best overall response of PR). When the best overall response is evaluated as SD, it must meet the protocol-specified minimum time from baseline. If the minimum time is not met, even if the best overall response is SD, the subject's best overall response will depend on the subsequent evaluation. For example, a subject who has SD at Cycle 1 and PD at Cycle 2 but does not meet the minimum duration for SD will have a best overall response of PD. The same subject who is lost to follow-up after SD in Cycle 1 will be considered inevaluable.

Best response determination in trials where confirmation of complete or partial response is required: Complete or partial responses may be claimed only if the criteria for each are met at a subsequent time point as specified in the protocol (generally 4 weeks later). In this case, the best overall response is described in Table 3.

4.4.4 Special notes on response assessment

When nodal disease is included in the sum of target lesions and the nodes decrease to "normal" size (< 10 mm), they may still have a measurement reported on scans. In order to avoid overestimation based on the increased size of the nodule, even if the nodule is normal, the measurement results will be recorded. As mentioned earlier, this means that subjects with CR may not have a total sum of zero on the CRF.

If confirmation of response is required during the trial, repeated "non-measurable" time points will complicate the best response assessment. The analysis plan for the trial must specify that these missing data/assessments can be clearly explained when determining efficacy. For example, in most trials, the response of PR-NE-PR in a subject can be considered as confirmed efficacy.

Symptomatic progression should be reported when a subject experiences an overall deterioration of health status requiring treatment discontinuation without objective evidence. Every effort should be made to assess objective progression even after treatment discontinuation. Symptomatic deterioration is not a descriptor of an objective response: it is the reason for stopping treatment. The objective response status of such subjects will be evaluated using the target and non-target lesions shown in Tables 1–3.

Conditions that define early progression, early death, and inevaluability are study specific and should be clearly described in each protocol (depending on treatment duration and treatment cycle).

In some cases, it may be difficult to distinguish local lesions from normal tissue. When the evaluation of complete response is based on such a definition, it is recommended that the biopsy be performed before the efficacy evaluation of local lesions for complete response is performed. When local radiographic abnormalities are considered representative of fibrosis or scarring in some subjects, FDG-PET should be used to confirm response to therapy in a manner similar to a biopsy. In such cases, the use of FDG-PET should be prospectively described in the protocol and supported by reports from specialist medical literature. However, it must be recognized that due to the limitations of FDG-PET and biopsy (including their resolution and sensitivity), false positive results will be obtained during CR evaluation.

For equivocal findings of progression (e.g., very small and uncertain new lesions; cystic changes or necrosis in existing lesions), treatment may continue until the next scheduled assessment. If at the next assessment, progressive disease is confirmed, the date of progression should be the date of the previous suspected progression.

4.5 Frequency of tumor re-evaluation

The frequency of tumor re-evaluation during treatment depends on the treatment regimen and should be consistent with the type and schedule of treatment. However, in phase II trials where the beneficial effect of treatment is unclear, follow-up every 6–8 weeks (timed to coincide with the end of a cycle) is reasonable, and the time interval can be adjusted under special protocols or circumstances. The protocol should specify which tissue sites should be evaluated at baseline (usually those most likely to be closely related to the metastatic lesion for the tumor type under study) and how often the evaluation should be repeated. Under normal circumstances, both target and non-target lesions should be evaluated at each assessment. In selected circumstances, certain non-target lesions may be evaluated less frequently. For example, a bone scan may need to be repeated only when the efficacy evaluation of the target disease is confirmed as CR or when progression of bone lesions is suspected.

After the end of treatment, tumor re-evaluation depends on whether the response rate or the time to an event (progression/death) is used as the endpoint of the clinical trial. If the time to an event (e.g., TTP/DFS/PFS) is the main endpoint of the study, then routine re-evaluation as specified in the protocol is required. In randomized comparative trials in particular, scheduled evaluations should be performed as outlined in the schedule (e.g., 6–8 weeks on treatment, or 3–4 months after treatment) and should not be affected by delays in treatment, dosing intervals, or any other events that may lead to imbalance in the treatment arm in the timing of disease evaluation.

4.6 Efficacy Assessment/Confirmation of Duration of Response

4.6.1 Confirmation

In non-randomized clinical studies where response is the primary endpoint, confirmation of PR and CR is required to ensure that responses are not the result of measurement error. This also allows for a reasonable interpretation of the results in the presence of historical data, but efficacy should also be confirmed in the historical data of these trials. However, in all other circumstances, such as in randomized trials (phase II or III) or studies where stable disease or progression are the primary endpoints, confirmation of response is not required since it will not add value to the interpretation of trial results. However, elimination of the requirement for response confirmation will prevent Central review of the effects of bias is more important, especially in non-blinded studies.

In the case of SD, measurements must have met the SD criteria at least once after study entry at a minimum interval (in general not less than 6–8 weeks) that is defined in the study protocol.

4.6.2 Overall response period

The duration of overall response is measured from the time measurement criteria are first met for CR/PR (whichever is first recorded) until the first date that recurrent or progressive disease is objectively documented (taking as reference for progressive disease the smallest measurements recorded on study). The duration of overall complete response is measured from the time criteria are first met for CR until the first date that recurrent or progressive disease is objectively documented.

4.6.3 Stable disease period

is measured from the start of the treatment until the criteria for PD are met (in randomized trials, from the date of randomization), taking as reference the smallest sum on study (if the baseline sum is the smallest, this is the reference for calculation of PD). The clinical relevance of the duration of stable disease varies by study and disease. If the proportion of patients achieving stable disease for a minimum period of time is an endpoint in a particular trial, the protocol should specify the minimal time interval required between two measurements in the definition of SD.

Note: The duration of response, stability, and PFS are affected by the frequency of follow-up after baseline evaluation. This definition of standard follow-up frequency does not fall within the scope of this guideline

circumference. The frequency of follow-up should take into account many factors, such as disease type and stage, treatment cycle, and standard practice. However, the accuracy limitations of these measured endpoints should be taken into account if comparisons between trials are to be made.

4.7 PFS/TTP

4.7.1 Phase II clinical trial

This guideline focuses primarily on the use of objective response as a study endpoint in phase II clinical trials. In some cases, the response rate may not be the optimal choice to evaluate the potential anticancer activity of a new drug/regimen. In these cases, PFS/PPF at landmark time points may be considered an appropriate surrogate for providing an initial signal of biological activity of the new agent. However, it is clear that in an uncontrolled trial, these assessments can be challenged because seemingly valuable observations may be related to biological factors such as patient selection rather than the effects of drug intervention. Therefore, phase II trials with these endpoints are best designed with randomized controls. However, the clinical presentation of some tumors is consistent (and usually consistently poor), and nonrandomized trials are reasonable. However, in these cases, it is important to document with care the evidence of efficacy when assessing the expected PFS or PPF in the absence of a positive control.
